# DNA methylation signatures of educational attainment

**DOI:** 10.1038/s41539-018-0020-2

**Published:** 2018-03-23

**Authors:** Jenny van Dongen, Marc Jan Bonder, Koen F. Dekkers, Michel G. Nivard, Maarten van Iterson, Gonneke Willemsen, Marian Beekman, Ashley van der Spek, Joyce B. J. van Meurs, Lude Franke, Bastiaan T. Heijmans, Cornelia M. van Duijn, P. Eline Slagboom, Dorret I. Boomsma, BIOS consortium, Bastiaan T. Heijmans, Peter A. C. ’t Hoen, Joyce van Meurs, Aaron Isaacs, Rick Jansen, Lude Franke, Dorret I. Boomsma, René Pool, Jenny van Dongen, Jouke J. Hottenga, Marleen MJ van Greevenbroek, Coen D. A. Stehouwer, Carla J. H. van der Kallen, Casper G. Schalkwijk, Cisca Wijmenga, Lude Franke, Sasha Zhernakova, Ettje F. Tigchelaar, P. Eline Slagboom, Marian Beekman, Joris Deelen, Diana van Heemst, Jan H. Veldink, Leonard H. van den Berg, Cornelia M. van Duijn, Bert A. Hofman, Aaron Isaacs, André G. Uitterlinden, Joyce van Meurs, P. Mila Jhamai, Michael Verbiest, H. Eka D. Suchiman, Marijn Verkerk, Ruud van der Breggen, Jeroen van Rooij, Nico Lakenberg, Hailiang Mei, Maarten van Iterson, Michiel van Galen, Jan Bot, Dasha V. Zhernakova, Rick Jansen, Peter van’t Hof, Patrick Deelen, Irene Nooren, Peter A. C. ’t Hoen, Bastiaan T. Heijmans, Matthijs Moed, Lude Franke, Martijn Vermaat, Dasha V. Zhernakova, René Luijk, Marc Jan Bonder, Maarten van Iterson, Patrick Deelen, Freerk van Dijk, Michiel van Galen, Wibowo Arindrarto, Szymon M. Kielbasa, Morris A. Swertz, Erik W. van Zwet, Rick Jansen, Peter-Bram’t Hoen, Bastiaan T. Heijmans

**Affiliations:** 1 Department of Biological Psychology, Vrije Universiteit Amsterdam, Amsterdam, The Netherlands; 2 Department of Genetics, University of Groningen, University Medical Centre Groningen, Groningen, The Netherlands; 3 Molecular Epidemiology section, Leiden University Medical Center, Leiden, The Netherlands; 4 Department of Epidemiology, Genetic Epidemiology Unit, Erasmus Medical Center, Rotterdam, The Netherlands; 5 Department of Internal Medicine, Erasmus Medical Center, Rotterdam, The Netherlands; 6 Department of Medical Statistics and Bioinformatics, Molecular Epidemiology Section, Leiden University Medical Center, Leiden, The Netherlands; 7 Department of Human Genetics, Leiden University Medical Center, Leiden, The Netherlands; 8 Department of Internal Medicine, ErasmusMC, Rotterdam, The Netherlands; 9 Department of Genetic Epidemiology, ErasmusMC, Rotterdam, The Netherlands; 10 Department of Psychiatry, VU University Medical Center, Neuroscience Campus Amsterdam, Amsterdam, The Netherlands; 11 Department of Genetics, University of Groningen, University Medical Centre Groningen, Groningen, The Netherlands; 12 Department of Biological Psychology, VU University Amsterdam, Neuroscience Campus Amsterdam, Amsterdam, The Netherlands; 13 Department of Internal Medicine and School for Cardiovascular Diseases (CARIM), Maastricht University Medical Center, Maastricht, The Netherlands; 14 Department of Gerontology and Geriatrics, Leiden University Medical Center, Leiden, The Netherlands; 15 Department of Neurology, Brain Center Rudolf Magnus, University Medical Center Utrecht, Utrecht, The Netherlands; 16 Department of Epidemiology, ErasmusMC, Rotterdam, The Netherlands; 17 Sequence Analysis Support Core, Leiden University Medical Center, Leiden, The Netherlands; 18 SURFsara, Amsterdam, The Netherlands; 19 Genomics Coordination Center, University Medical Center Groningen, University of Groningen, Groningen, The Netherlands; 20 Department of Medical Statistics and Bioinformatics, Medical Statistics Section, Leiden University Medical Center, Leiden, The Netherlands

## Abstract

Educational attainment is a key behavioural measure in studies of cognitive and physical health, and socioeconomic status. We measured DNA methylation at 410,746 CpGs (*N* = 4152) and identified 58 CpGs associated with educational attainment at loci characterized by pleiotropic functions shared with neuronal, immune and developmental processes. Associations overlapped with those for smoking behaviour, but remained after accounting for smoking at many CpGs: Effect sizes were on average 28% smaller and genome-wide significant at 11 CpGs after adjusting for smoking and were 62% smaller in never smokers. We examined sources and biological implications of education-related methylation differences, demonstrating correlations with maternal prenatal folate, smoking and air pollution signatures, and associations with gene expression in cis, dynamic methylation in foetal brain, and correlations between blood and brain. Our findings show that the methylome of lower-educated people resembles that of smokers beyond effects of their own smoking behaviour and shows traces of various other exposures.

## Genetics: DNA methylation as a marker of education

DNA methylation, one of the epigenetic marks in cells, is associated with educational achievement (EA). A team led by Jenny van Dongen from the Vrije Universiteit Amsterdam tested more than 400,000 sites across the entire genome for their relationship with educational achievement in 4152 Dutch adults. 58 such sites were found that were located in and near genes with neuronal, immune and developmental functions. DNA methylation signatures of EA revealed differential exposure to cigarette smoke, even after accounting for own smoking behaviour, and differential exposure to folate and air pollution. EA predicts differential life conditions, including life expectancy. This study shows that these conditions leave their traces in the methylome of white blood cells and affect gene expression. Future research may investigate how these genes influence individual differences in behaviour, education, and health.

## Introduction

Educational attainment correlates across time and countries with prosperity, and across individuals within populations with cognitive functioning,^1^ personality,^2^ physical and mental health,^3^ and socioeconomic conditions across the lifespan.^4^ A recent genome-wide association study (GWAS) reported an association between educational attainment and SNPs at 74 loci.^5^ The findings pointed at biological pathways that contribute to individual differences in educational attainment in human populations: A large proportion of SNPs was associated with gene expression in the foetal brain and the functions and expression patterns of the loci pointed at an involvement in neural development processes. Throughout development and the adult lifespan, gene expression is regulated by epigenetic mechanisms, such as DNA methylation. DNA methylation plays a crucial role in brain development and learning,^6,7^ and is increasingly recognized as an important mechanism that mediates genetic and environmental effects on health. Changes in DNA methylation are a key characteristic of cancer,^8^ and have been implicated in diseases including obesity,^9^ type 2 diabetes,^10,11^ depression,^12^ and Alzheimer’s disease.^13^ In addition to genetic variation, variation in DNA methylation between individuals exists as a result of stochastic factors and environmental influences.^14–17^ In humans and animals, various early life exposures can induce stable long-term changes in DNA methylation.^18–20^ Examples include early postnatal maternal behaviour,^20^ childhood abuse,^19^ and prenatal maternal nutrition, the effects of which are still detectable in blood cells at middle-age.^18^ Later life exposures also induce changes to the methylome, for example exposure to cigarette smoke.^21,22^

Educational attainment is linked to numerous environmental conditions across the lifecourse,^23^ including prenatal, childhood and adult nutrition, healthcare, neighbourhood environment and occupation, many of which are connected to (behavioural) characteristics that also show heritable variation between individuals, such as smoking, stress, alcohol use and BMI. These exposures are thus potential candidate drivers of differential epigenetic patterns between higher and lower educated people. Furthermore, to the extent that these exposures elicit epigenetic responses in relevant tissues at key (developmental) periods in life, epigenetic mechanisms may play a role in mediating causal effects of these exposures on (developmental) pathways related to cognitive, physical and mental health.

Educational attainment is strongly associated with socioeconomic status.^4^ The epigenetic causes and effects of social status have been well-characterized in various animal species.^24–27^ Social status in female rhesus macaques can be manipulated experimentally through order of introduction of the animal in a group, and has widespread effects on genome-wide DNA methylation and gene expression in white blood cells^26^ and immune functions, with low-status promoting pro-inflammatory and antibacterial responses and high-status promoting anti-viral responses.^28^ Previous small to moderately sized human studies (average sample size = 439, range = 40–1264) have reported associations between measures of childhood or adulthood socioeconomic status or parental education and DNA methylation levels at individual genome-wide sites,^29–31^ candidate genes related to inflammation, stress, cardiometabolic risk and the insulin pathway^32–37^ and global DNA methylation,^38,39^ though others did not find a significant relationship with global DNA methylation.^40^ A few studies have examined DNA methylation in blood in relation to cognitive performance. A study of children reported associations between methylation levels of CpGs in *HES1* in cord blood with IQ at age 4 years (*N* = 175), executive memory function at age 7 (*N* = 200), and externalizing behaviour at age 1 (*n* = 108).^41^ A study in 168 adults from the Barbados Nutrition Study examined DNA methylation in relation to attention (ADHD index) and IQ^42^ and identified 134 differentially methylated regions (DMRs) associated with severe protein-energy malnutrition in early postnatal life. Methylation level of three of these CpGs also correlated with IQ and CpGs in 13 of the DMRs were also associated with attention. Thus far, one study has examined DNA methylation at individual loci in relation to educational attainment.^43^

We performed a large association study of educational attainment and genome-wide DNA methylation in whole blood (Illumina 450k array) in 4152 adult participants from four population-based cohorts from across the Netherlands, born between 1925 and 1989. During this period, educational opportunities have greatly improved in the Netherlands, especially for women. We took these trends into account by analysing birth cohort- and sex-specific education scores derived by ridit transformation of the highest completed level of education.^44^ Even though the Netherlands currently is one of the wealthiest and most highly educated countries in the world, significant health differences exist between higher and lower educated Dutch people: The difference in average life expectancy at birth is 6 to 7 years between men and women with and without a higher education,^45^ and 14 years when considering lifespan without physical limitations.^45^ The aim of the current study was to identify loci where DNA methylation level is associated with educational attainment in blood. Such loci may point to epigenetic consequences of differential life conditions that correlate with educational attainment (i.e. epigenetic biomarkers of exposure), peripheral correlates of the epigenetic mechanisms that contribute to individual differences in educational attainment for instance by regulating gene expression in neurons and thereby affecting brain development or later cognitive functioning, and peripheral epigenetic correlates of education-related health differences (which may be biomarkers or may be part of the causal mechanisms that contribute to disease, respectively). To examine the extent to which different environments contribute to education-related differences in DNA methylation, we interpret our results against information on the heritability of DNA methylation levels across age, the relationship between DNA methylation and educational attainment within adult twin pairs who grew up in the same household, and summary statistics from previously published epigenome-wide association studies of various lifestyle factors and exposures. To interpret biological implications, we test the association between DNA methylation level at education-associated CpGs and transcript levels and perform network analysis. Finally, to enable inferences about the implications of our findings to brain biology, we employ previously published methylomic datasets to study the variation displayed by education-associated CpGs across foetal brain development^46^ and the correlation of their methylation levels between blood and brain tissue.^47^

## RESULTS

### Study characteristics and birth cohort trends in educational attainment

Characteristics of participants of each biobank (NTR; *N* = 2172, LLS; *N* = 668, RS; *N* = 608, and LLD; *N* = 704) are described in Table 1. The mean birth year of participants was 1968 in NTR, 1945 in LLS, 1943 in RS, and 1965 in LLD. Educational attainment showed a strong, sex-specific, increase across birth cohorts (supplemental Fig. 1). These trends reflect changes in educational opportunities that have taken place in the Netherlands in this time period, due to increasing wealth and changes in societal attitude towards pursuing a higher degree of education, and changes in the compulsory number of years of education. To obtain a standardized measure of educational attainment, the seven categories were transformed into sex- and birth cohort-specific ridit scores, which reflect an individual’s educational position within his or her birth cohort. This measure reflects the social value of a certain level of education within society at a given time, and captures individual differences in educational attainment when individuals from different birth cohorts and sexes are simultaneously analysed. In the entire dataset, ridit scores were strongly correlated with educational attainment level (women: *r* = 0.82, men: *r* = 0.93, supplemental Fig. 2), but they are, by definition, standardized for sex and birth cohort differences.

**Table 1:** Participant characteristics from 4 population-based cohorts in the Netherlands

Cohort	NTR	LLS	RS	LLD
N samples	2199^a^	668	608	704
% female	68.6	52.5	57.7	57.7
Mean age at blood sampling (sd)	38.2 (12.6)	59.1(6.6)	68.6 (5.6)	47.3 (12.5)
Mean BMI (sd)	24.4 (4.0)	25.5 (3.5)	27.7 (4.2)	25.5 (4.2)
Mean birth year (range)	1968(’26-’89)	1945 (’25-’74)	1943 (‘30-‘60)	1965(’31-’87)
Smoking status (%)				
Current smoker	19.2	12.7	9.4	18.3
Former smoker	24.3	55.5	55.3	35.8
Never smoker	56.5	31.7	35.4	45.3
Educational attainment (%)				
1. Primary school only	2.7	3.9	15.8	1.1
2. Lower vocational schooling	7.6	22.3	20.7	8.4
3. Lower secondary schooling (general)	7.1	18.3	15.8	11.1
4. Intermediate vocational schooling	28.2	19.2	23.7	24.3
5. Intermediate/higher secondary schooling (general)	5.6	3.1	4.1	9.2
6. Higher vocational schooling	27.9	23.7	17.6	32.4
7. University	20.8	9.6	2.3	13.5

*NTR* Netherlands Twin Register, *LLS* Leiden Longevity Study, *RS* Rotterdam Study, *LLD* Lifelines-Deep, *Sd* Standard deviation

^a^From 2172 participants (for 27 subjects, DNA methylation data from two longitudinal peripheral blood samples were included, collected with an interval of on average 5 years)

### Meta-analysis identifies 58 significant CpGs

**Fig. 1:**
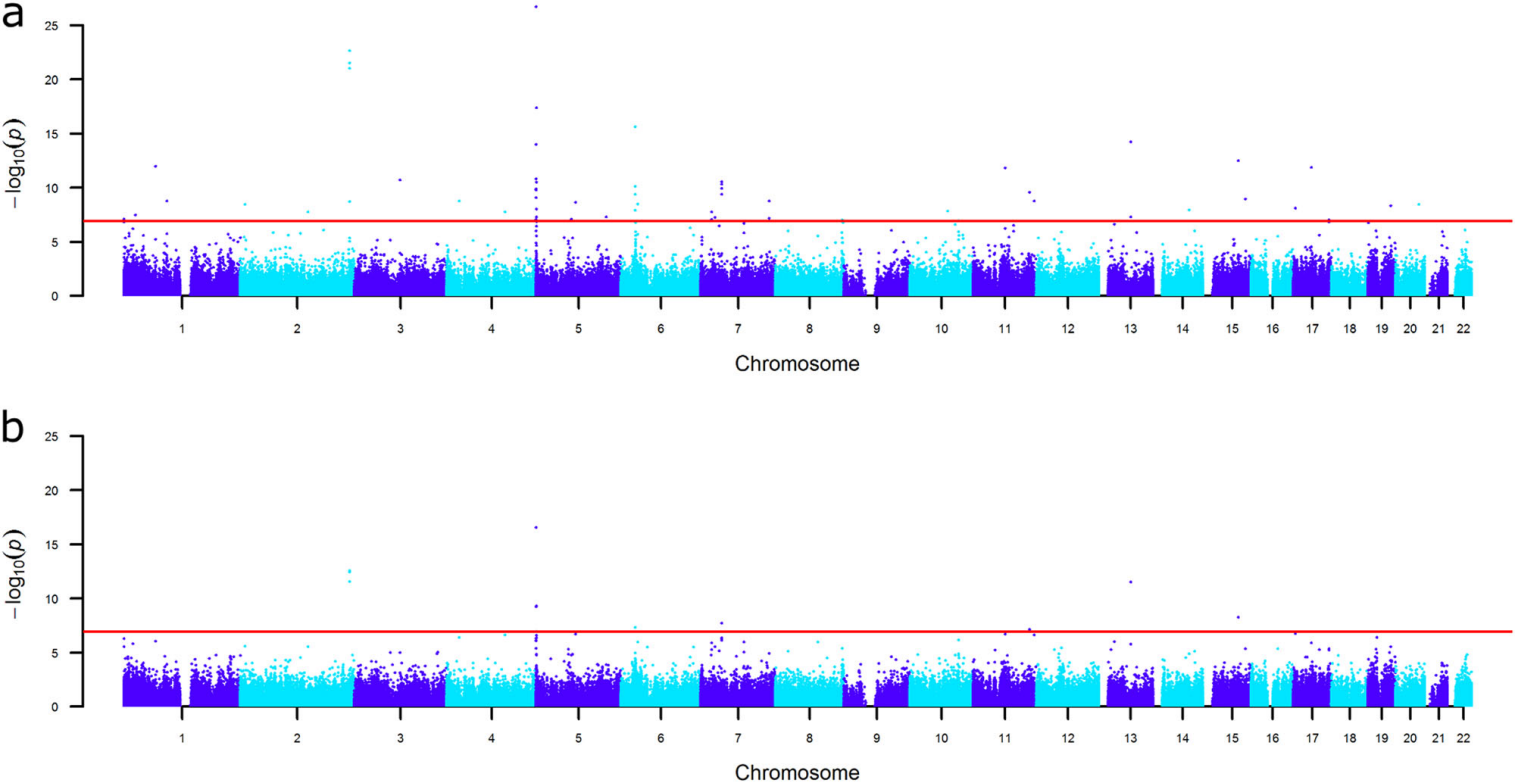
Manhattan plot showing the fixed effects meta-analysis p-values for the association between educational attainment and DNA methylation level. **a** EWAS not adjusted for individual smoking status. The red line denotes the significance threshold. **b** EWAS adjusted for individual smoking status. The red line denotes the significance threshold

Genome-wide DNA methylation analyses were performed within each cohort to test the association between the standardized educational attainment score and DNA methylation level, while adjusting for age, sex, white blood cell counts and technical covariates. Results were combined in a fixed effects meta-analysis adjusting for test-statistics bias and inflation (Bayesian estimates of bias and inflation from all analyses are provided in Supplemental Table 1). The meta-analysis identified 58 significant CpG sites (*P* < 1.2 × 10^−7^, Bonferroni correction for 410,746 tests) mapping to 35 genes (Fig. 1a; Supplemental Table 2). The number of genome-wide significant sites in individual cohorts and in the meta-analysis is presented in Supplemental table 3. The meta-analysis effects sizes (betas) range from a difference in methylation percentage of 0.6 to 6.0% between the lowest value (0) and the highest value (1) of education on the ridit scale. At 44 sites (76%), a higher education level was associated with a higher methylation level.

Several CpG sites map to genes that have been previously linked to cognitive or behavioural phenotypes. For example, cg23126342 (beta = −0.03, se = 0.004, *P* = 6.1 × 10^−15^) and cg25491122 (beta = −0.01, SE = 0.003, *P* = 5.6 × 10^−8^) map to *PCDH9* on chromosome 13, encoding protocadherin 9. Copy-number variants in this gene have been linked to autism spectrum disorder^48^ and gene knock-out in mice causes social and object recognition deficits.^49^ *PCDH9* has also been described as a tumor-suppressor gene.^50^ Two significant sites are located in *CNTNAP2* on chromosome 7, encoding Contactin Associated Protein-Like 2; a neuronal transmembrane protein part of the neurexin family that mediates cell–cell interaction (cg25949550: beta = 0.009, se = 0.002, *P* = 1.8 × 10^−9^; and cg21322436: beta = 0.01, se = 0.002, *P* = 6.7 × 10^−8^). *CNTNAP2* is regulated by forkhead box protein P2 (*FOXP2*);^51^ a transcription factor with a crucial role in language development.^52^ Genetic variants in *CNTNAP2* have been linked to several neurodevelopmental syndromes and conditions including language impairment, autism, intellectual disability, dyslexia, epilepsy, Tourette syndrome and schizophrenia.^53^ Other interesting top hits include cg06695691 in *SPATA5* (mutations in this gene cause microcephaly, intellectual disability, seizures and hearing loss,^54^ but the gene has also been associated with hair loss^55^ and was first described for its role in spermatogenesis^56^), cg08549335 in *ZNRF2*, encoding a protein involved in neuronal transmission and plasticity,^57^ cg18181703 in *SOCS3*; encoding a key regulator of cytokine signalling, and cg00310412 in *SEMA7A*, encoding semaphorin 7 A. This is a membrane-bound member of the semaphorin family of guidance proteins and is involved in signalling pathways in the immune and nervous system (stimulating axon outgrowth).^58^

Of all 58 significant CpGs, 7 are located within 500 kb of a SNP with a nominal p-value ( < 1 × 10^−4^) in the currently largest published GWAS of educational attainment,^5^ including 4 CpGs in *MYO1G*, cg21611682 in *LRP5*, cg11827514 (intergenic chromosome 8, nearest gene *MIR4472-1*) and cg15857661 (*MIR146B*). None of the SNPs associated with cognitive functions (reaction time and verbal numerical reasoning) based on the GWAS in UK Biobank^59^ were located within 500 kb of our significant EWAS hits.

Of all CpG sites associated with educational attainment, CpGs in genes that are known to be linked to cognition-related phenotypes are likely candidates for being involved in causal epigenetic mechanisms that contribute to individual differences in educational attainment, provided that these loci also show education-associated differences in methylation in the relevant tissue (most likely, the brain). Overlap between GWAS loci and EWAS loci could imply that genetic variation and DNA methylation variation at these loci both contribute to variation in educational attainment or that genetic variants for educational attainment influence DNA methylation. Yet, it is also possible that DNA methylation differences reflect education-associated lifestyle differences.

### Methylation hits educational attainment overlap with signatures of exposure to cigarette smoke

The site with the lowest p-value is cg05575921 (beta = 0.06, se = 0.006, *P* = 2.1 × 10^−27^), located in the *AHRR* gene on chromosome 5, which codes for the Aryl-Hydrocarbon Receptor Repressor. AHRR is a negative regulator of the Aryl-Hydrocarbon Receptor (AhR); a ligand-activated transcription factor that regulates the expression of many genes in many tissues. The best characterized (exogenous) ligands of AhR are environmental contaminants from the polycyclic aromatic hydrocarbon (PAH) family such as benzo[a]pyrene (B[a]P), and halogenated aromatic hydrocarbons (HAHs) such as TCDD (dioxin).^60^ The AhR pathway is well-described as a detoxification pathway since AhR regulates the expression of many genes encoding enzymes for xenobiotic metabolism, but is also involved in normal development and cell functions,^61^ and is activated by various endogenous ligands, such as kynurenine and other metabolites of tryptophan.^60^ Many of the long-recognized toxic effects of dioxins and other environmental contaminants, including birth defects, carcinogenicity, disruption of endocrine and reproductive systems, immune dysregulation and neurotoxicity, are mediated by the AhR pathway.^62^ Therefore, it may be speculated that this pathway plays a role in education-related cognitive and health differences.

**Fig. 2:**
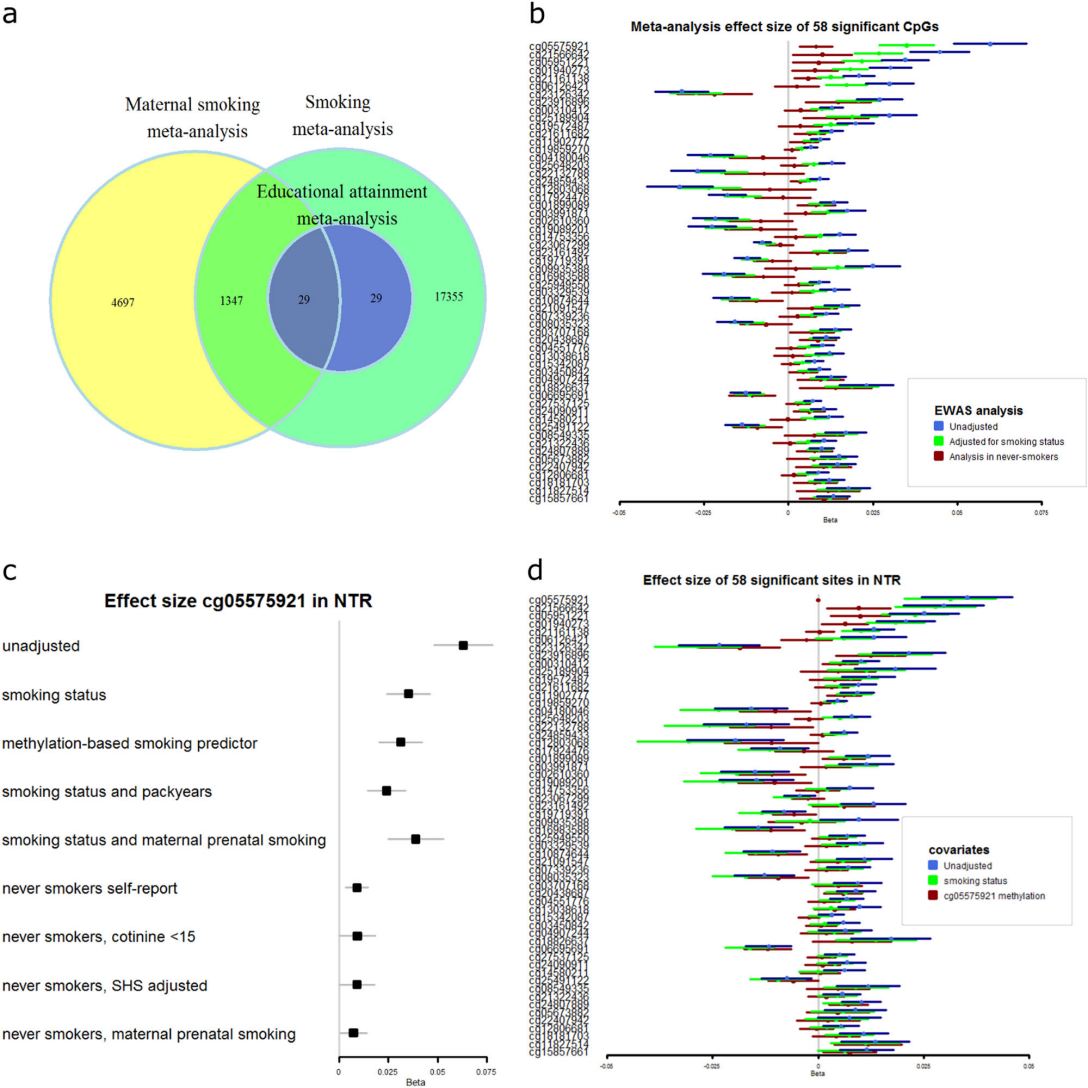
Overlap of educational attainment signatures and cigarette smoke signatures. **a** Venn-diagram showing the overlap between methylation sites associated with educational attainment in our meta-analysis and methylation sites previously reported to be associated with individual smoking or with maternal prenatal smoking in cord blood at birth. **b** Forest plot showing educational attainment meta-analysis effect sizes (betas) of significant CpGs, with and without adjustment for smoking status, and in never-smokers. **c** Educational attainment effect sizes (betas) in the NTR cohort for educational attainment top site cg05575921 (*AHRR*) while adjusting for various covariates, in all NTR subjects and in never smokers. SHS = second hand smoke exposure, indexed by plasma cotinine levels between 1 and 15. **d** Forest plot showing educational attainment fixed effects meta-analysis effect sizes (betas) of significant CpGs, with and without adjustment for self-reported smoking status, and adjusting for methylation level at cg05575921 (*AHRR*). The error bars display 95% confidence intervals. The sizes of squares are scaled by the size of the confidence intervals

Our top site, cg05575921, is one of the strongest associated and most consistently reported sites in human EWA studies of phenotypes related to cigarette smoke exposure.^63^ We overlaid all 58 sites significantly associated with educational attainment with sites of which the methylation level has been previously reported to be associated with individual smoking (18,760 sites^21^) or prenatal maternal smoking (6073 sites^64^) in large meta-analyses and observed large overlap (Fig. 2a): all educational attainment associated sites are also associated with individual smoking and 29 (50%) are currently known to be associated with maternal prenatal smoking (Supplemental Table 2). Educational attainment and smoking behaviour are negatively correlated (*r* = −0.11, *p* = 6.3 × 10^−12^ for current smoking, r = −0.09, *p* = 1.5 × 10^−9^ for ever smoking, correlations for each birth cohort separately are provided in Supplemental table 4). Therefore, the effect size of the association between educational attainment and DNA methylation level in adults will incorporate (confounding) effects of the individual’s smoking behaviour at loci affected by exposure to cigarette smoke. Mediation analysis showed that the association between educational attainment and methylation was partially mediated by smoking and the proportion of effect mediated varied across the 58 top sites, from 7 to 47% (average 28%, Supplemental Table 2). To account for confounding effects, we next tested the relationship between educational attainment and DNA methylation after adjusting for smoking (i.e. considering the direct effect in the association between education and methylation) and when restricting the analysis to never-smokers. These analyses will inevitably also take out relevant variation related to educational attainment (and reduce sample size when restricting to never-smokers).

After adjusting for smoking status, the effect size for the association between educational attainment and methylation level at cg05575921 (*AHRR*) was attenuated by 41% but still highly significant (beta = 0.035, se = 0.004, *p* = 3.0 × 10^−17^; Supplemental Fig. 3). In total, 11 CpGs were genome-wide significant while adjusting for smoking status (*P* < 1.2 × 10^−7^, Fig. 1b, Supplemental Table 2. The 11 sites with genome-wide significant direct effects of educational attainment are characterized by varying degrees of mediation by smoking (13–42%).

**Fig. 3:**
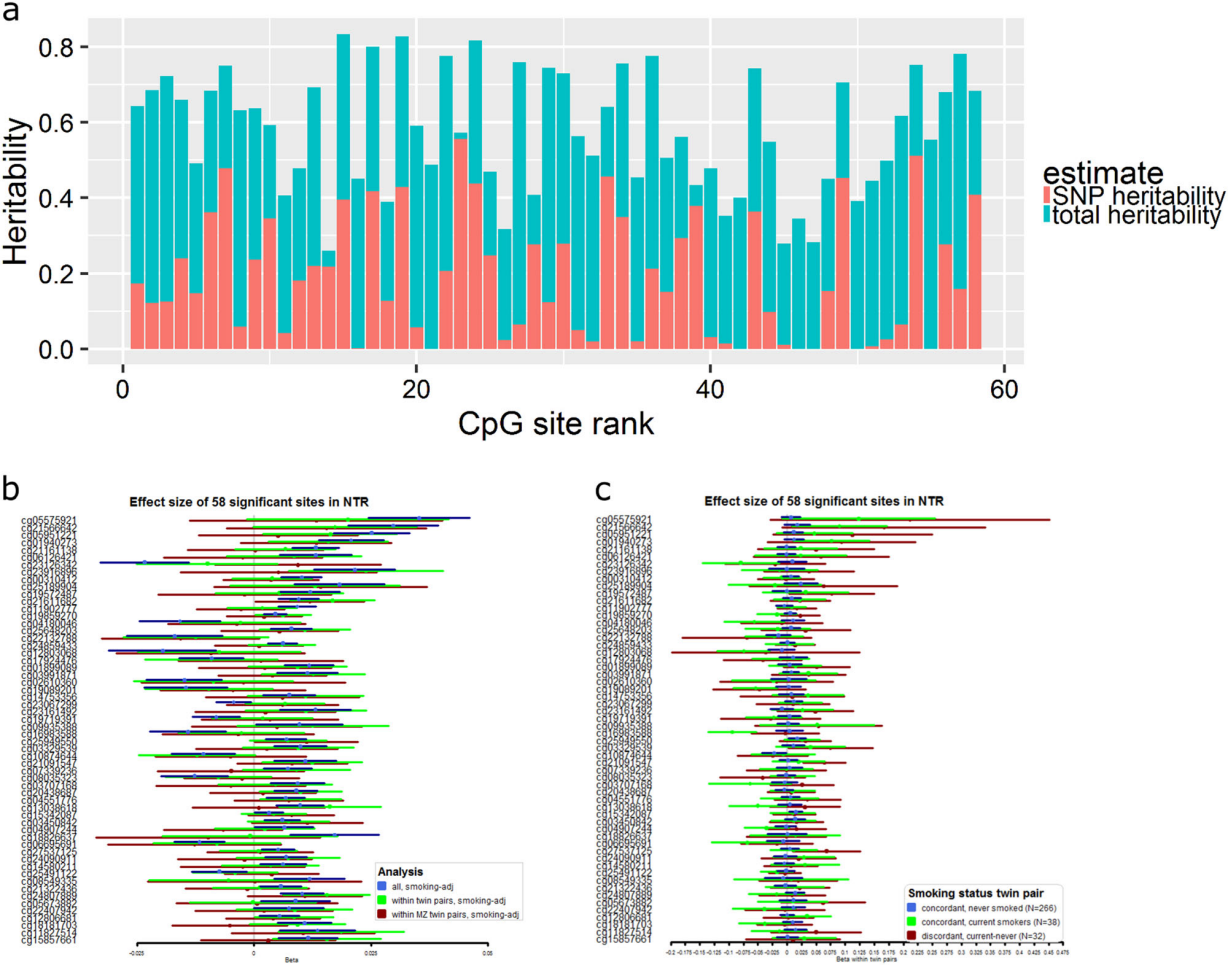
Heritability of CpGs associated with educational attainment and relationship between educational attainment and methylation level in twins. **a** Total heritability and proportion of variance explained by all genome-wide SNPs. **b** The forest plot shows the effect sizes (betas) of the 58 top sites for educational attainment adjusted for smoking status: 1) in the entire NTR cohort and 2) within twin pairs, derived by regressing the within-pair methylation difference on the within-pair educational attainment difference. **c** The forest plot shows the effect sizes (betas) of the 58 top sites for educational attainment in smoking discordant twin pairs (current smoker and never smoker) and smoking concordant twin pairs (both twins current smokers or both twins never smoked)

After exclusion of all current smokers and former smokers (52% of the sample) from the analysis (New *N* = 2001), the meta-analysis effect size for the association between educational attainment and methylation at cg05575921 (*AHRR*) was 86% smaller compared to its initial effect size (beta = 0.008, SE = 0.003, *p* = 1.3 × 10^−3^, Fig. 3b). This is comparable to the effect size of CpG sites in *CNTNAP2 (*ranked 31 and 51 in our EWAS). Across all 58 CpG sites, the effect sizes in never-smokers were on average 60% smaller than in the entire dataset. Examples of CpGs for which the effect sizes in never-smokers were most similar to those in the entire population are cg23126342 in *PCDH9* (beta = −0.02, se = 0.006, *p* = 1 × 10^−4^ in never smokers), cg06695691 in *SPATA5* (beta = −0.01, se = 0.004, *p* = 2 × 10^−4^ in never smokers), and cg04907244 in *SNORD93* (beta = 0.01, se = 0.004, *p* = 7 × 10^−4^ in never smokers). These sites are characterized by small indirect effects of education in the entire population (proportion of total effect mediated by smoking = 13% for cg23126342, 8% for SPATA5 and 15% for SNORD93, respectively). For these sites, we thus see strongest evidence that methylation level correlates with educational attainment beyond effects of own smoking behaviour; however, we note that when taking all 58 top sites, effect sizes in never-smokers were strongly correlated with effect sizes in the entire population (r = 0.83), were in the same direction at 57 sites, and were at least nominally significant in never-smokers (*p* < 0.05) at 27 sites (Fig. 2b). Based on the effect size of the top 58 sites in the current study, we performed a power analysis to estimate the required sample size to detect associations between methylation and educational attainment in never smokers with 80% power at genome-wide significance (alpha = 1 × 10^−7^; supplemental Fig. 4). This analysis shows that 3735 never smokers would be required for the CpG with the largest effect (cg23126342, *PCDH9*, percentage of variance explained = 1% in never smokers).

We hypothesized that residual confounding by smoking may explain (part of) the association between educational attainment and methylation after adjusting for smoking. We examined smoking misclassification effects by considering two biomarkers of smoking in NTR: a methylation-based smoking score and plasma cotinine levels. Adjusting for a methylation-based smoking score overall had similar effects as adjusting for self-reported smoking status (Supplemental Table 5, Fig. 2c), although effect sizes for educational attainment were slightly smaller at many CpGs, confirming a small effect of smoking misclassification. Plasma cotinine levels were available for 591 individuals classified as never smokers by self-report. Five of these individuals (0.8%) had cotinine levels > = 15 ng/mL, which is indicative of smoking, and thus indicates a misclassification of smoking status^65^). After excluding these individuals from the analysis of never smokers (new *N* = 586), effect sizes for educational attainment were similar for 9 of the 11 CpGs that are genome-wide significant for educational attainment after adjusting for smoking (r = 0.68, concordant direction of effect = 82%, Fig. 2c, Supplemental Table 5). The strongest associated CpG was cg23126342 in *PCDH9* (beta = −0.03, se = 0.009, *p* = 3 × 10^−4^ in never smokers with cotinine < 15 ng/mL). These analyses illustrate that associations between methylation and educational attainment are to some extent affected by smoking misclassification, but that associations between educational attainment and methylation remain when accounting for objective measures of smoking.

To examine whether smoking intensity, number of years smoked, and prenatal smoking exposure may account for part of the association between education and methylation, we performed further analyses in NTR, considering two other smoking-related phenotypes; pack-years and exposure to maternal smoking during pregnancy. Here, we describe our education top site cg05575921 (Fig. 2c). The results for all sites are given in Supplemental table 5. In NTR, smoking pack-years was independently associated with methylation level at cg05575921 (*N* = 2786, beta = −0.002, se = 0.0004, *p* = 7.2 × 10^−10^) on top of the effect of individual smoking status (beta = −0.07, se = 0.003, *p* = 2.6 × 10^−111^). When pack years was added (in addition to smoking status) to the model testing the relationship between educational attainment and methylation level at cg05575921, the association between educational attainment and methylation level was further attenuated but still present (*N* = 2056, beta = 0.02,se = 0.005, *p* = 7.1 × 10^−7^). We analyzed information on maternal smoking during pregnancy that was available for 1506 NTR subjects (mean age = 34, frequency of exposed = 10%) while adjusting for individual smoking status. In line with previous reports in babies and children,^64^ maternal smoking during pregnancy was associated with lower methylation at cg05575921 in this adult population while adjusting for individual smoking status (beta = −0.04, se = 0.008, *p* = 1.7 × 10^−6^), and also with methylation level at many of the other education top sites that were previously found to be associated with maternal smoking in newborns (strongest association with maternal smoking: cg25949550, *CNTNAP2*, beta = −0.017, se = 0.002, *p* = 1.50 × 10^−15^, Supplemental Table 6). In NTR, maternal smoking during pregnancy was not associated with maternal educational attainment (*N* = 545, beta = −0.023, se = 0.041, *p* = 0.584), or with offspring educational attainment (*N* = 1269, beta = −0.028, se = 0.030, *p* = 0.35). Adjustment for maternal smoking in NTR had little effect on the association between educational attainment and methylation at education top sites. The effect size for educational attainment at cg05575921 remained unchanged after adjusting for prenatal maternal smoking in all individuals (*N* = 1243, beta = 0.04, se = 0.007, *p* = 6.2 × 10^−8^) and was slightly reduced in the group of non-smoking individuals (*N* = 770, beta = 0.007, se = 0.003, *p* = 0.03).

We examined effects of current second-hand smoking exposure using information on serum cotinine levels in never smokers in NTR (*N* = 586). This analysis included never smokers with cotinine levels < 1 mg/mL (*N* = 436, 74%) and never smokers with cotinine levels > = 1 and < 15 ng/mL, which is indicative for second-hand smoking exposure^65^ (*N* = 150, 26%). After adjusting for second-hand smoke exposure (1–15 ng/mL) when testing the association between educational attainment in never smokers, associations in the same direction and with similar effect size were obtained for 10 out of 11 education top sites (r = 0.86, Supplemental Table 5), including cg05575921 in *AHRR* (*N* = 586, beta = 0.009, se = 0.005, *p* = 0.05 in never smokers adjusting for current second-hand smoking exposure, Fig. 2c).

Finally, we evaluated within NTR the effect of adjusting for methylation level at cg05575921 (*AHRR*), as an indicator of lifetime exposures that interact with the aryl hydrocarbon receptor (AhR) pathway, including lifetime smoke exposure and toxins from sources other than cigarette smoke and endogenous substrates (e.g. kynurenine) mediated by this pathway. Adjustment for cg05575921 methylation level profoundly reduced the effect size for the majority of the 58 significant hits for educational attainment (Fig. 2d). Yet, for 51 CpGs (88%) the effect was still in the same direction, the effect sizes with and without adjusting for cg05575921 methylation level were strongly correlated (r = 0.79), and 21 were at least nominally significant (*p* < 0.05, *N* = 2172). Most strongly associated with educational attainment after adjusting for cg05575921 were cg06695691 in *SPATA5* (beta = −0.012, SE = 0.003, *p* = 2.71 × 10^−5^) and cg23126342 in *PCDH9* (beta = −0.019,SE = 0.005,*p* = 1.5 × 10^−4^). The results for the association with educational attainment after adjusting for pack-years, maternal smoking during pregnancy, and cg05575921 methylation are reported for all 58 sites in Supplemental Table 5. Adjustment for body-mass-index (BMI), as an indicator of lifestyle factors, had little impact on the effect sizes of our top sites (Supplemental Table 2).

### Heritability of top hits for education

To gain further insight into the sources of variation in DNA methylation between individuals at education-associated CpGs, we overlaid them with information from a previous study where we estimated variance in DNA methylation explained by genetic effects (heritability) and environmental effects, and the interaction of these effects with age and sex in adults (mean age = 37).^17^ The 58 pGs associated with educational attainment were characterized by a moderate to high heritability (range = 0.26 - 0.83, mean = 0.58) and an average SNP heritability (proportion of variance explained by genome-wide SNPs) of 0.19 (range: 0–0.56, Fig. 3a), illustrating that genetic variation contributes to methylation variation at education-associated loci. The variance explained by individual-specific environmental and stochastic effects (“unique environment”) for these sites was on average 0.42. At 19 CpGs (33%), the unique environmental variance increased significantly with increasing age (*P* < 1.2 × 10^−7^), including CpGs annotated to *AHRR, ALPPL2, HLA-DRB5, GNG12, RARA, LRP5, MYO1G, TBC1D19, YWHAQ, SNORD93 and MIR4472-1*, leading to a decrease in the heritability of these sites with increasing age. At 1 CpG (near *ZEB2*) the additive genetic variance increased significantly with increasing age (*P* < 1.2 × 10^−7^). None of the education-associated CpGs showed a significant difference in the genetic or environmental variance between men and women, a significant common environmental component, or a significant non-additive genetic component. The moderate to high heritability of education-associated CpGs together with their known sensitivity to exposure to cigarette smoke may indicate that heritable variation at these loci (partly) reflects heritable variation in behaviour that gives rise to being exposed (e.g. smoking behaviour), or that effects of relevant exposures (e.g. cigarette smoke) on methylation depend to a certain extent on genotype. Methylation levels may also be an intermediate mechanism of genetic variants (methylation QTLs) associated with educational attainment (independently of any environmental exposure).

### Relationship between educational attainment and DNA methylation differences in twins

Because twins start their lives in the same womb, grow up in the same family environment and live together at least until they leave their parental home, they are largely matched for prenatal and childhood exposures. Variation in childhood socioeconomic environment and childhood exposures (e.g. prenatal and postnatal second-hand exposure to cigarette smoke, diet etc.) are thus controlled for by design when analysing the relationship between differences in DNA methylation and educational attainment between co-twins. This analysis also controls for part of the genetic variation in the population. We tested the association between DNA methylation differences at the 58 top sites and differences in educational attainment in 1478 twins (mean age = 37, including 492 monozygotic and 247 dizygotic pairs), after adjusting for smoking status. The effect sizes within twin pairs correlated strongly with those across all people (r = 0.83). The observation that the association between educational attainment and DNA methylation is also present within families shows that the association in the entire population is not merely driven by population stratification effects (of genetic or environmental origin). The effect sizes within twin pairs were on average 0.76 of the effect sizes in the entire population (beta within twin pairs divided by beta in all subjects). Especially the highest ranking CpG sites for education showed smaller effect sizes in the within-twin pair analysis (Fig. 3b). If own smoking behaviour would be the sole cause for methylation changes associated with educational attainment, differences in educational attainment between twins are expected to be associated with similar methylation differences as observed in relation to education differences in the entire population. When restricting to monozygotic twins only, effect sizes were further reduced (Fig. 3b) to on average 0.28 of the effect sizes in the entire population (r = 0.49). This pattern suggests that, at these loci, at least part of the association between methylation and educational attainment in the population that remains after adjusting for individual smoking status is driven by factors in the environment that are shared by twins or by a shared genetic influence on educational attainment and DNA methylation.

Next, we analysed the relationship between within-pair differences in DNA methylation and educational attainment separately in smoking concordant and smoking discordant monozygotic twin pairs (Fig. 3c). The effect sizes were largest in smoking discordant monozygotic twins (*N* = 32 pairs, note that we did not adjust for smoking status in this analysis), followed by concordant current smoking twins (*N* = 38 pairs). Effect sizes were smallest within concordant twin pairs of which both twins never smoked (*N* = 266 pairs). These effect sizes were also generally smaller compared to the effects sizes observed in all never smokers (median ratio = 0.7, where ratio = beta within non-smoking concordant monozygotic twin pairs/beta in all non-smokers).

### Correlation with summary statistics from previous EWASs of individual smoking, maternal smoking, maternal folate, air pollution and alcohol consumption

**Fig. 4:**
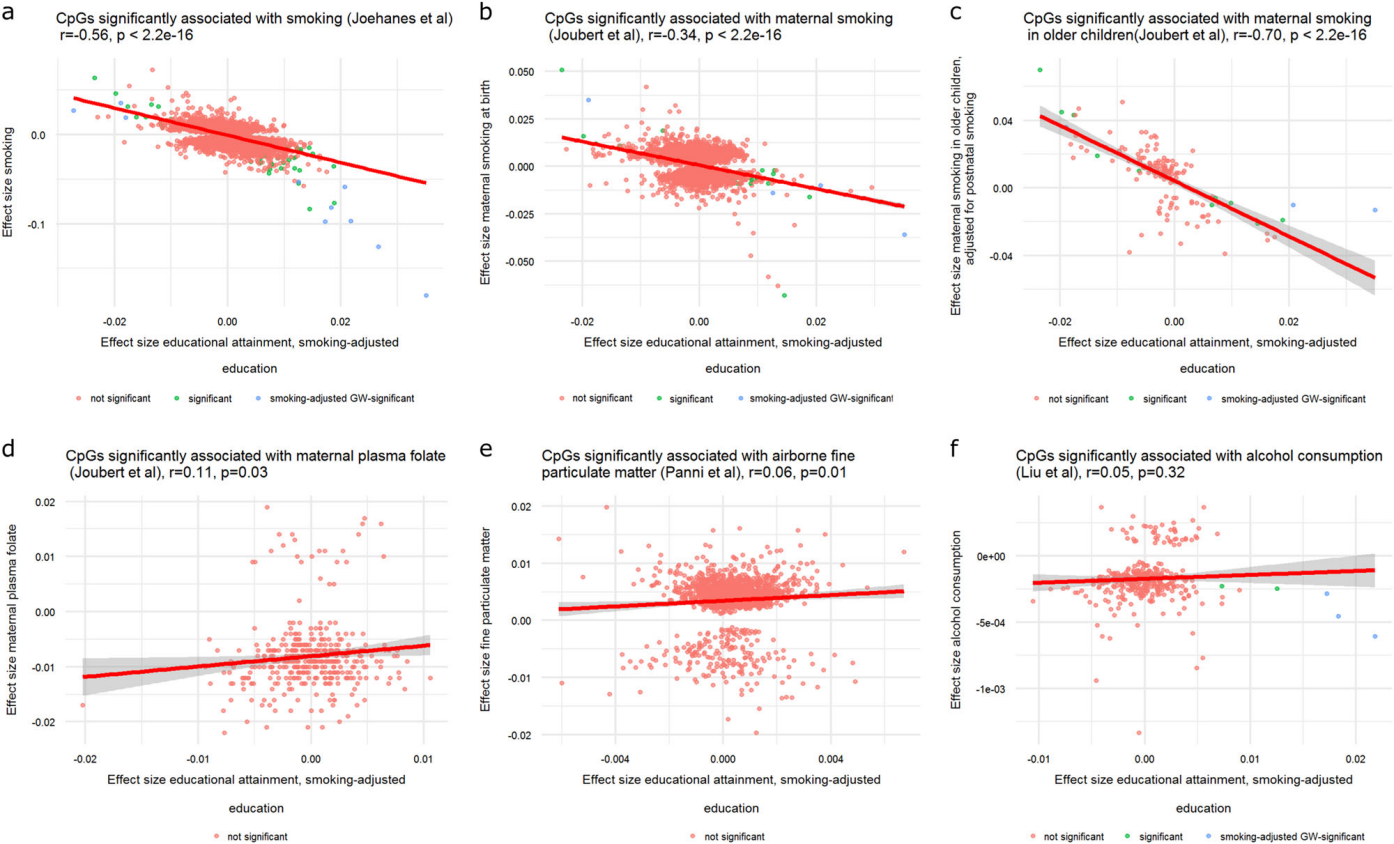
Pearson correlation between effect sizes from previous epigenome-wide association studies and the meta-analysis effect sizes for educational attainment. **a** Methylation sites significantly associated with smoking. The effect sizes for smoking (current minus never smoking, y-axis) are plotted against the effect sizes (betas) for educational attainment, adjusted for smoking status (x-axis). **b** Methylation sites significantly associated with maternal smoking at birth. The effect sizes for maternal smoking in cord blood at birth (exposed minus non-exposed babies, y-axis) are plotted against the effect sizes (betas) for educational attainment, adjusted for smoking status (x-axis). **c** Methylation sites significantly associated with maternal smoking in older children. The effect sizes for maternal smoking in older children (exposed minus non-exposed children, y-axis) are plotted against the effect sizes (betas) for educational attainment, adjusted for smoking status (x-axis). **d** Methylation sites significantly associated with maternal plasma folate level. The effect sizes for maternal plasma folate level in cord blood at birth (y-axis) are plotted against the effect sizes (betas) for educational attainment, adjusted for smoking status (x-axis). **e** Methylation sites significantly associated with 28-day average fine matter particulate concentration. The effect sizes for fine particulate matter concentration in the environment (y-axis) are plotted against the effect sizes (betas) for educational attainment, adjusted for smoking status (x-axis). **f** Methylation sites significantly associated with alcohol intake. The effect sizes for alcohol intake (y-axis) are plotted against the effect sizes (betas) for educational attainment, adjusted for smoking status (x-axis). GW-significant Genome-wide significant

To examine the resemblance between methylomic signatures of education and methylomic signatures of various environmental exposures, we computed the correlation between the effect size (beta or mean methylation difference between groups) from previous EWA-studies of various exposures and effect size (beta) from our EWAS meta-analysis of educational attainment (Fig. 4). Methylation sites significantly associated with smoking according to the EWAS meta-analysis of smoking^21^ (and present in our EWAS, *N* = 16,932 sites) showed differences in methylation proportion ranging from −0.18 to 0.07 (current minus never smokers) and education-related differences ranging from −0.03 to 0.06 (highest minus lowest value of educational attainment) without adjustment for smoking status, and −0.03 to 0.04 adjusted for smoking status. The correlation between effect sizes was r = −0.74 taking the education EWAS results without adjustment for smoking status, and r = −0.56 for the education EWAS with adjustment for smoking status (Fig. 4a).

Methylation sites significantly associated with prenatal maternal smoking^64^ and present in our EWAS (*N* = 5575), showed effect sizes ranging from −0.07 to 0.05 in relation to prenatal maternal smoking (in cord blood at birth) and −0.02 to 0.04 in relation to educational attainment in adults (adjusted for individual smoking status). The correlation between effect sizes of maternal smoking at birth and educational attainment was negative (r = −0.34, *p* < 2.2 × 10^−16^, Fig. 4b), and stronger (r = −0.70, *p* < 2.2 × 10^−16^, Fig. 4c) when restricting to 144 sites that still showed significant differences in older children (average age 6.8) in the previous maternal smoking meta-analysis after adjusting for postnatal second-hand smoking exposure; indicative of persistent effects of maternal smoking. Of the CpGs with persistent maternal smoking effects, 13 were also significant in our meta-analysis of educational attainment. Maternal plasma folate level^66^ (N CpGs = 419, r = 0.11, *p* = 0.03, Fig. 4d) and 28-day average concentration of airborne fine particulate matter^67^ (N CpGs = 1578, r = 0.06, *p* = 0.01, Fig. 4e) showed small positive correlations with educational attainment. None of the CpGs associated with maternal folate level or concentration of fine particulate matter were genome-wide significant in our meta-analysis of educational attainment. Effect sizes of sites significantly associated with alcohol consumption^68^ did not correlate significantly with educational attainment effect sizes (*N* = 344, r = 0.05, *p* = 0.3, Fig. 4f). Five sites were significantly associated with educational attainment and significantly associated with alcohol intake: cg03329539, cg05951221 and cg01940273, located intergenic on chromosome 2 (nearest gene = *ALPPL2*), cg06126421, located intergenic on chromosome 6 (nearest gene = *HLA-DRB5*) and cg19572487 in *RARA*. At these 5 CpGs, methylation level correlates positively with educational attainment and negatively with alcohol intake.

### DNA methylation is associated with gene expression in cis

We tested the association between DNA methylation at education-associated CpGs and RNA levels in cis ( < 100 kb) using data from 3002 whole blood samples from the BIOS consortium with data on DNA methylation and RNA-seq. At 33 CpGs (57%), methylation level was associated (*P* < 1.1 × 10^−4^, Bonferroni correction for 463 tests) with the levels of one or multiple transcripts (total N significant associations = 58, Supplemental Table 7). This analysis confirmed a relationship between methylation level and expression level of the same gene for *AHRR*, *RARA*, *GPR15, MYO1G*, *ANPEP*, *ATP9A* and *SOCS3*, revealed associations between intergenic CpGs and nearby transcripts (e.g. intergenic CpGs on chromosome 6 were associated with expression level of *FLOT1* and *NRM*), and associations between methylation level and multiple transcripts within a region. Several education-associated CpGs in *AHRR* were associated with the level of *AHRR* transcripts and other transcripts, including *EXOC3*, which codes for exocyst complex component 3, a component of a multiprotein complex involved in vesicle trafficking in polarized epithelial cells and neural synapses. Methylation level at cg05575921 (*AHRR*) and *AHRR* transcript level correlated negatively (ENSG00000063438, beta = −4.5 (log Counts Per Million per methylation proportion), se = 0.23, *p* = 1.03 × 10^−81^).

### Network analysis

Network analysis in GeneMANIA, employing information on genetic and protein-protein interactions, co-expression, co-localization, pathway information, and shared protein domains indicated that all but 2 of the genes (*TMEM136* and *TTLL10*) annotated to the 58 CpG sites associated with educational attainment are connected to each other as part of a bigger network of co-expressed or interacting genes (Supplemental Fig. 5a). For example, genetic interactions have been identified between *PCDH9* and *CNTNAP2, ZNRF2 and ZMIZ1* and protein-protein interactions occur between *CNTNAP2* and *ZMIZ1*. Networks based on transcript levels that were significantly associated with methylation level of education-CpGs, and based on genes that harbour education-associated CpGs whose methylation levels did not show significant associations with transcript levels in blood are illustrated Supplemental Figs. 5b and 5c.

### Methylation at education-associated CpGs in foetal and adult brains

**Fig. 5:**
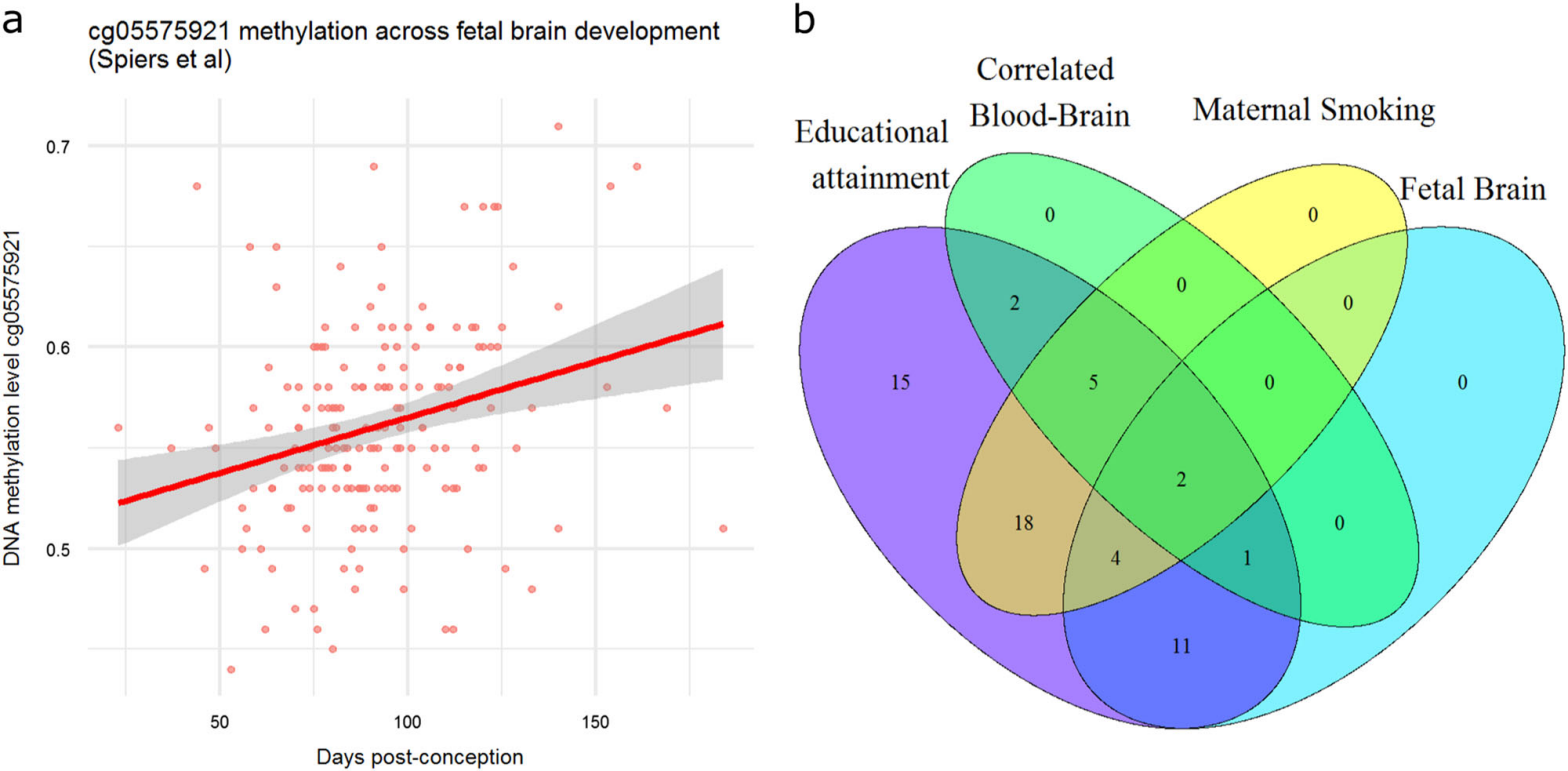
Methylation level in the brain during foetal development at cg05575921 and the number of educational attainment top sites displaying significant changes in foetal brain, significant correlations between blood and brain, or significant associations with maternal smoking. **a** Methylation level in brain tissue (y-axis) is plotted against foetal age (x-axis) for cg05575921 (*AHRR*). **b** Venn-diagram for the overlap between educational attainment associated sites, sites significantly correlated between blood and one or more brain tissues, sites associated with maternal smoking (based on the previously published meta-analysis by Joubert et al.)^64^ and sites where methylation level in the brain correlates significantly with foetal age. All significant sites for educational attainment are included in the figure (*N* = 58).

To examine the implications of our findings to brain biology, we looked up the methylation level of our 58 top CpGs in the brain using two previously published Illumina 450k datasets: one study assessed DNA methylation levels over the course of human foetal brain development (a period that was linked previously to the action of SNPs associated with educational attainment^5^) and the other study assessed DNA methylation levels in whole blood and four brain regions from matched post-mortem samples consisting of prefrontal cortex, entorhinal cortex, superior temporal gyrus and cerebellum.^47^ At 18 of our top CpGs for educational attainment (31%), DNA methylation shows changes in the foetal brain as a function of foetal age (r = −0.57 to r = 0.38, *p* < 7.7 × 10^−4^, Bonferroni correction for 58 tests, Supplemental Table 8), which may indicate that methylation of these CpGs plays a role in foetal brain development. This includes 6 sites associated with prenatal maternal smoking, including our top site for educational attainment, cg05575921 in *AHRR* (r = 0.27, *p* = 2.5 × 10^−4^, Fig. 5a*)*. At 10 of our top CpGs (17%), methylation level in blood correlates significantly with DNA methylation level in at least one out of four assessed brain regions, with correlations ranging from r = 0.36 to r = 0.63 (*P* < 2 × 10^−4^; Bonferroni correction for 232 tests, Supplemental Table 9). This implies that variation in methylation level at these CpGs between adult individuals in blood is to a certain extent reflective of variation of their methylation level in the brain. The strongest correlated CpG is cg05951221 (chromosome 2 intergenic, nearest gene *ALPPL2*, r = 0.63, *p* = 1.1 × 10^−9^ between blood and superior temporal gyrus; and r = 0.55, *p* = 3.8 × 10^−7^ between blood and prefrontal cortex). The overlap of CpGs associated with educational attainment and maternal smoking effects, methylation changes in foetal brain and correlations between blood and brain is illustrated in Fig. 5b. We highlight 2 examples of CpGs, which are implicated by all 4 analyses: cg23161492 in *ANPEP* (a gene previously identified in the GWAS of Type 2 diabetes^69^ and implicated in major depressive disorder by pathway analysis^70^) and cg04907244 in *SNORD93*, a snoRNA that was previously reported as a potential early diagnostic marker of Alzheimer’s disease because it was found to be differentially expressed in juvenile cortices of a mouse model for Alzheimer’s disease.^71^ Both CpGs were also associated with gene expression levels in cis in blood (Supplemental Table 7).

## Discussion

By employing an epigenome-wide association approach in a large consortium study of adult subjects from the Netherlands with harmonized data for educational attainment, we have identified methylation sites significantly associated with educational attainment. Importantly, these results indicate that methylation levels at many top CpGs correlate with educational attainment beyond the effects of a person’s own smoking behaviour, although a large part of the effect size of top loci for educational attainment correlated with the current and past smoking behaviour of individuals. Our main conclusion is supported by the outcomes of three distinct analyses. Firstly, educational attainment is associated with DNA methylation after adjusting for smoking status (11 CpGs were genome-wide significant after Bonferroni correction) and pack-years. Secondly, the association was present in individuals who never smoked in their lives and in smoking-concordant twin pairs. Thirdly, the effect size was smaller within twin pairs compared to the effect size in the entire population. This suggests that factors in the childhood (or prenatal) environment or genetic factors may account for part of the association between educational attainment observed in the entire population. Residual confounding effects of smoking might explain part of the association between DNA methylation and educational attainment that remains after adjusting for smoking phenotypes (e.g. smoking status may be misclassified or smoking variables may not fully capture effects of lifelong smoking on DNA methylation dynamics),^72^ but analyses of data on cotinine levels and methylation-based smoking prediction scores showed that associations remained when accounting for objective biomarkers of individual smoking. The analyses in never-smokers strongly suggested that education captures variation in DNA methylation related to sources other than own smoking behaviour. In summary, methylation sites that are most strongly associated with educational attainment in blood point to epigenetic consequences of differential exposures that correlate with educational attainment, of which own smoking behaviour explains a large but not entire part.

During the review process of this manuscript, another large EWAS of educational attainment was published (N=10767 subjects from 27 cohorts from multiple countries).^43^ This study identified 9 CpGs after adjusting for smoking, of which 8 were also among the top 58 CpGs from the current study.

Multiple scenarios may explain the relationships among smoking, DNA methylation and educational attainment observed in this study that can be roughly summarized as follows: 1) DNA methylation is merely a biomarker of differential exposures between higher and lower-educated people, but does not play a role in the causal chain of biological mechanisms that influence educational attainment or smoking, 2) genetic variants have independent effects on educational attainment, smoking and DNA methylation (genetic pleiotropy), which may induce a correlation among these outcomes even if none of the outcomes has a causal effect on the others, 3) DNA methylation in blood marks epigenetic mechanisms in the brain that influence educational attainment and/or smoking. In the third scenario, DNA methylation mediates downstream causal effects of genetic variants and/or exposures (e.g. smoking) on cognitive and behavioural outcomes by influencing gene expression. In scenarios 1 and 3, exposure to smoke may either involve exposure via the individual’s own smoking behavior or via second-hand exposure (e.g. prenatal exposure to parental smoking, postnatal second-hand exposure in the family household, public areas, at work etc.).

To investigate a potential causal effect of DNA methylation on educational attainment or smoking behavior, the relevant question that remains to be answered is: if we could change DNA methylation at these loci, would this result in a change in cognitive performance or (smoking) behaviour? One approach to address this question is by Mendelian randomization analysis, involving a test of whether genetic variants that influence DNA methylation at these loci are also associated with cognitive and behavioural outcomes. Such studies may test specific biological hypotheses through the use of genetic variants that affect DNA methylation in specific cell types (i.e. neuronal cells from various regions of the brain) and life stage (e.g. foetal development, childhood, adolescence/young adulthood). Importantly, the genetic variants should explain a sufficient proportion of the variation in DNA methylation to perform a well-powered analysis, and genetic pleiotropy should be ruled out or controlled for (i.e. the genetic variants should not influence educational attainment or smoking through mechanisms other than via methylation of the CpG site in question). Causality modeling such as Mendelian randomization can also shed light on the question whether smoking has causal effects on educational attainment and DNA methylation. Furthermore, data collected before and after the introduction of smoke-free legislation (e.g. smoking bans in workplaces and/or public places) may be used to examine the question if reducing exposure to (second-hand) smoking might increase educational attainment, in the same way that smoke-free legislation has been reported to lead to reductions in preterm births, hospital attendance for asthma,^73^ and reduced incidence of low birth weight.^74^

We found differential methylation at genes involved in neuronal, immune and developmental processes. Larger EWA and GWA studies of educational attainment will be valuable to obtain a more detailed picture of the overlap between loci detected in genetic versus epigenetic studies. Among loci where methylation changes are merely biomarkers of education-related exposures or lifestyle we do not expect to see enrichment of GWAS loci of educational attainment. If at least some of the EWAS associations, however, are due to a causal effect of epigenetic regulation on educational attainment, it would be expected that the overlap between loci from GWAS and EWAS is larger than expected by chance. Based on what is currently known about the functions of the genes identified in this study and the traits and diseases they have been implicated in, these findings can have implications for studies into brain development, cognitive and mental health, cancer, inflammatory disease and reproductive health.^49,50,53–57,61,62,70,71^ Cohorts with DNA methylation data in children may answer the question whether methylation level at these loci is associated with cognitive functioning in childhood and whether they predict future educational attainment of a child. Cohorts with detailed information on pregnancy characteristics such as PACE^64^ may provide more insight into the role of maternal behaviour and exposures during pregnancy. In the current study, we did not have objective measures of maternal smoking during pregnancy available such as prenatal cotinine levels.

Our results illustrate that there is a clear education-related gradient in cigarette smoke exposure signatures in the methylome, even in individuals who never smoked. An important next question is whether (part of) the observations in peripheral blood are reflective of a correlated or systemic epigenetic response to cigarette smoke or a related exposure across tissues, with unfavourable effects in the brain. A large body of literature exists on the associations between prenatal maternal smoking, childhood second-hand exposure and individual smoking and health outcomes, cognitive functions and mental health. Findings in support of a causal effect of exposure to cigarette smoke on mental health have been reported, for example for the link between prenatal smoking and child externalizing behaviour,^75^ individual smoking during adolescence and attention,^76^ and individual smoking and schizophrenia.^77^ Earlier findings indeed lend support for the hypothesis that epigenetic regulation of some of our top loci may affect brain development: i.e. 1) genetic variants in several of our top loci are known to cause neurodevelopmental phenotypes (including for example autism and intellectual disability),^48,53,54^ or the role of the gene in brain functions has been characterized in functional studies^49,57^; 2) cigarette smoking-associated DNA methylation changes in peripheral blood were reported to be enriched near genes associated with educational attainment and schizophrenia (and many other diseases and traits), identified through GWAS^21^; 3) disruption of aryl hydrocarbon receptor signalling by prenatal or postnatal lactational exposure to dioxin (a component of cigarette smoke) disturbs dendritic growth in the hippocampus and amygdala of mice.^78,79^ Together with our results these findings advocate further investigation on whether exposure to cigarette smoke prenatally, in childhood or adolescence may cause adverse effects on the developing or mature brain and what other exposures may affect methylation of the genes highlighted by our meta-analysis.

The AhR pathway is well-known to be sensitive to many combustion-derived and synthetic environmental contaminants including PAHs and HAHs, some of which are present in cigarette smoke. These compounds are ubiquitous in the environment and accumulate in food, water, soil, and air due to their long biological half-lives. Other potential exposures are a variety of naturally occurring plant-derived dietary compounds that are known to activate or inhibit the AhR pathway.^60^ The correlations between previously published effect sizes of EWASs and effect sizes from our meta-analysis of educational attainment give insight into education-dependent traces of various exposures in the methylome. These correlations show, in addition to education-dependent traces of cigarette smoke, a small positive correlation between educational attainment and prenatal maternal folate level, suggestive of methylomic traces of higher folate levels in higher educated people. The small positive correlation between effect sizes associated with airborne fine matter particle concentration and educational attainment effect sizes suggests that higher educated people in our study show more traces of exposure to ambient fine matter particles, which could reflect the correlation in this population between a higher level of education and living in urban areas. Maternal prenatal folate levels and airborne fine particulate matter concentration do not appear to account for the association of our top hits with educational attainment, since these CpGs were not significantly associated with maternal prenatal folate or fine matter.

Importantly, the implications of our findings also depend on the extent to which DNA methylation of these loci in blood is a marker for epigenetic regulation in the tissues and life stages where variation in cognitive functions and health arises. We have obtained some insight regarding the methylation levels of our top sites in the brain through look-up in previously published datasets of methylation in brain tissues. This analysis showed that individual differences in methylation level in blood are significantly correlated with individual differences in brain regions at 10 sites. At 18 sites, DNA methylation level in the brain changes as a function of foetal age, which may imply a role in brain development.

Strengths of this study include the fact that phenotype harmonization, DNA methylation and RNA measurements, data processing and QC was performed for all samples together in a harmonized way at one facility as part of the BIOS consortium. In addition, this is the largest study thus far investigating DNA methylation in relation to educational attainment or related phenotypes. The homogeneity of the study population and their educational system has both strengths and limitations. All subjects were from the Netherlands and such homogeneity of the study population, both with respect to phenotyping and genetic background, maximizes power to detect methylation sites associated with educational attainment. Yet, further studies in other populations are warranted to examine the generalizability of these findings across subjects from different countries, with different educational systems, levels of prosperity and lifestyles. A limitation is that despite our large sample size, even larger samples would be required to detect the effect sizes we observed in never smokers at genome-wide significance. Our top site in never smokers explains 1% of the variance in educational attainment in never smokers and would require a sample size of 3735 individuals for 80% power at an alpha of 1 × 10^−7^ (genome-wide significance following Bonferroni correction). The rest of the top 10 sites require sample sizes between ~5000 and 7500 never smokers. Larger international collaborations with appropriate phenotyping are required to detect these sites at genome-wide significance. Another limitation is that we only had access to blood samples (as is often the case in EWA studies) from adult subjects. It is expected that DNA methylation levels in blood samples from adults are more informative about the effects of lifelong conditions and exposures related to educational attainment than about the most important epigenetic determinants of cognitive abilities. However, this does not rule out that our findings may turn out to be informative peripheral epigenetic markers of educational attainment-related exposures or to be informative about a subset of biological pathways that contribute to variation in cognitive abilities and may be monitored in peripheral blood. Previous EWASs of diseases that are thought to affect primarily the brain, such as Alzheimer’s disease and schizophrenia illustrate that cognitive decline later in life and psychiatric diseases with a developmental component are, to a certain extent, associated with DNA methylation changes in peripheral blood at loci that show similar methylation changes in the brain (Alzheimer’s disease^80^) and loci known to be causally involved in disease pathology (e.g. schizophrenia GWAS genes).^81^

## METHODS

### Cohorts

The EWAS was performed with data from 4152 participants from four population-based Dutch cohorts participating in the Biobank-based Integrative Omics Study (BIOS) consortium,^82,83^ covering subjects from all regions of the Netherlands: the Netherlands Twin Register (NTR),^84^ the Leiden Longevity Study (LLS),^85^ the Rotterdam Study (RS)^86^ and LifeLines DEEP (LLD).^87^ Genome-wide methylation in whole blood was measured for all samples by the Human Genotyping facility (HugeF) of ErasmusMC, the Netherlands (http://www.glimdna.org/). Data on educational attainment, body mass index (BMI), smoking status, white blood cell counts and DNA methylation were available for the following number of samples: 2199 samples from 2172 individuals (NTR), 668 (LLS), 608 (RS), 704 (LLD).

### Ethics approval and consent to participate

The study was approved by the institutional review boards of the participating centers (LLD, Ethics committee of the University Medical Centre Groningen; LLS, Ethical committee of the Leiden University Medical Center; NTR, Central Ethics Committee on Research Involving Human Subjects of the VU University Medical Centre; RS, Institutional review board (Medical Ethics Committee) of the Erasmus Medical Center). All participants have given written informed consent and the experimental methods comply with the Helsinki Declaration.

### Educational attainment

Educational attainment was defined as the highest completed level of education at the age of 25 or higher. It was classified on a 7-point scale: 1 = primary school only, 2 = lower vocational schooling, 3 = lower secondary schooling (general), 4 = intermediate vocational schooling, 5 = intermediate/higher secondary schooling (general), 6 = higher vocational schooling, 7 = university. The raw 7-category educational attainment level data were transformed into ridit scores (“relative to an identified distribution”, Supplemental Methods).^44^

### Peripheral blood DNA methylation and cell counts

DNA methylation was assessed with the Infinium HumanMethylation450 BeadChip Kit (Illumina, San Diego, CA, USA). Genomic DNA (500 ng) from whole blood was bisulfite treated using the Zymo EZ DNA Methylation kit (Zymo Research Corp, Irvine, CA, USA), and 4 μl of bisulfite-converted DNA was measured on the Illumina 450 k array^88^ following the manufacturer’s protocol. A custom pipeline for quality control and normalization of the methylation data was developed by the BIOS consortium. First, sample quality control was performed using MethylAid.^89^ Next, probe filtering was applied to remove: ambiguously mapped probes,^90^ probes with a detection *P* value > 0.01, or bead number < 3, or raw signal intensity of zero. After these probe filtering steps, probes and samples with a success rate < 95% were removed. Next, the DNA methylation data were normalized using functional normalization,^91^ as implemented in *minfi*,^92^ using the cohort-specific optimum number of control probe-based principal components. Probes containing a SNP, identified in a DNA sequencing project in the Dutch population,^93^ within the CpG site (at the C or G position) were excluded irrespective of minor allele frequency and only autosomal probes were analysed. The EWAS was performed on 410746 methylation sites. For all samples included in the EWAS, white blood cell counts were measured with the standard white blood cell differential as part of the complete blood count (CBC).

### Smoking phenotypes

In all cohorts, smoking status was defined according to 3 categories: current smoker, former smoker, never smoker. Information on plasma cotinine levels were analysed in NTR. Plasma cotinine level measurements have been described previously.^65^ We used previously described cut-offs to classify smokers (cotinine > = 15 ng/mL) and individuals exposed to second-hand smoking (cotinine levels > = 1 and < 15 ng/mL).^65^ Relationships between DNA methylation level and smoking pack-years, prenatal maternal smoking and a methylation-based smoking score were studied in NTR. Pack-years were calculated as the (number of cigarettes smoked per day/20) × number of years smoked. Information on number of cigarettes smoked per day and number of years smoked was obtained as part of the NTR biobank project protocol at the moment of blood draw. We analysed information on maternal smoking during pregnancy that was obtained in NTR Survey 10 (data collection in 2013) with the following question: Did your mother ever smoke during pregnancy? with answer categories: no, yes, I don’t know. For twin pairs, the answers were checked for consistency and missing data for one twin were supplemented with data from the co-twin where possible: If one twin answered “yes” or “no”, and his/her co-twin did not fill out the survey, or did not answer this question, or answered “I don’t know”, the missing information was replaced by the information supplied by the co-twin. If one twin answered “yes” and the co-twin answered “no” (3.6% of twin pairs for which an answer was available for both twins), data from both twins were set to missing. A methylation-based smoking score was calculated in NTR using the same methods as described in^94^ and applied in a recent EWAS of schizophrenia.^95^ This method takes the methylation levels at sites previously reported to be associated with smoking status to produce a weighted score, using the weights from a previous smoking EWAS. We used the same weights as applied in,^94,95^ which are based on the smoking EWAS by Zeilinger et al.^96^

### Statistics

#### EWAS

All statistical analyses were performed in R.^97^ The association between DNA methylation level and educational attainment was tested under a linear model with DNA methylation β-value as outcome. Three models were tested. The simplest model (model 1) included the following predictors: educational attainment, sex, age at blood sampling, HM450k array row, bisulphite plate (dummy-coding) and white blood cell percentages (% neutrophils, % monocytes, and % eosinophils in NTR, LLS and LLD; %monocytes and % granulocytes in RS). Additional models were fitted to test whether the association between DNA methylation and educational attainment is attenuated by adjusting for body-mass-index (BMI; as a correlate of lifestyle factors) or smoking status (current smoker, former smoker or never smoked). Model 2 included the same predictors as model 1 plus smoking status, and model 3 included the same predictors as model 1 plus BMI and smoking. Finally, model 1 was also run after excluding all current and former smokers. In NTR, generalized estimation equation (GEE) models were fitted with the R package gee. The following settings were used: Gaussian link function (for continuous data), 100 iterations, and the “exchangeable” option to account for the correlation structure within families. In LLS, RS, and LLD linear regression analyses were performed with the R function lm(). The EWAS analyses were performed separately in each cohort. Next, results were combined in a fixed effects meta-analysis adjusting for test-statistics bias and inflation using the method described by van Iterson et al.^98^ as implemented in the R-package Bacon. We applied stringent Bonferroni correction for the number of methylation sites tested (alpha = 0.05/410746 = 1.2 × 10^−7^).

#### Follow-up analyses

Follow-up analyses, including mediation analysis, power analysis, relationships with a methylation-based smoking score, pack-years, and prenatal smoking during pregnancy, analysis of twin data, correlations with previously published summary statistics, analyses of previously published brain datasets and network analysis are described in the Supplemental Methods.

#### Association with gene expression in cis

The association between DNA methylation and transcripts in cis was tested using data from 3002 samples from 6 cohorts from the BIOS consortium for which whole blood DNA methylation and RNA-seq data were available (NTR, RS, LLS, LLD, the Prospective ALS Study Netherlands (PAN), and the Cohort on Diabetes and Atherosclerosis Maastricht (CODAM)).^82,83^ We selected transcripts within 100 kb of education-associated CpGs, excluding the 1% least variable transcripts and transcripts for which a read was detected in < 1% of the samples. For one CpG, no transcripts were detected within 100 kb that met these criteria (total N CpGs = 57, total N methylation-transcript associations tested = 463). For each CpG, we performed a transcriptome-wide association analysis in each cohort. Transcript levels (log counts per million) were regressed on methylation levels (beta), and the following covariates: age, sex, % monocytes, % lymphocytes, % neutrophils, bisulphite plate, and Hiseq2000 flowcell. In LLS, bisulphite plate was not included as covariate because it was highly correlated with flowcell. Missing cell count data were imputed as previously described.^99^ Results were combined in a fixed effects meta-analysis. To adjust for test-statistics bias and inflation, the transcriptome-wide association analysis was performed using the method described by van Iterson et al.^98^ with the R-packages CATE (5-factors) and Bacon. We applied stringent Bonferroni correction for the number of tests performed (alpha = 0.05/463 = 1.1 × 10^−4^).

### Annotations

To identify overlap between our EWAS results and results of the GWAS of educational attainment by Okbay et al.^5^ we obtained the pruned list of 5000 SNPs (EduYears_Pooled_5000.txt) from http://ssgac.org/ and selected all SNPs with a p-value < 1 × 10^−4^ for educational attainment in the meta-analysis of all discovery and replication cohorts (1681 SNPs). We also checked for overlap with SNPs significantly associated with cognitive functions (reaction time and verbal numerical reasoning) in the GWAS in UK Biobank.^59^ Methylation sites previously associated with individual smoking or prenatal exposure to maternal smoking at a false-discovery rate (FDR) < 0.05 were obtained from recent large meta-analyses by Joehanes et al.^21^ and Joubert et al.^64^ Finally, we obtained methylation sites associated with maternal prenatal folate level at FDR < 0.05,^66^ air pollution at FDR < 0.05 (defined as 28-day average fine particulate matter concentration measured daily at fixed monitoring stations in Germany and the US),^67^ and alcohol consumption (defined as a continuous trait) at Bonferroni genome-wide significance in Europeans.^68^

### Data availability

The HumanMethylation450 BeadChip and RNA-seq data described in this paper are available in the European Genome-phenome Archive (EGA), under the accession code EGAD00010000887. The genome-wide results from this EWAS are available here: http://bbmri.researchlumc.nl/atlas.

## Electronic supplementary material

supplemental material(DOCX 2707 kb)

Supplemental Tables 1-9(XLSX 95 kb)

## Electronic supplementary material

**Supplementary information** accompanies the paper on the *npj Science of Learning* website (10.1038/s41539-018-0020-2).
