# Supplementary material for: DNA methylation signatures of educational attainment
Source: NPJ Sci Learn. 2018 Mar 23;3:7. doi: 10.1038/s41539-018-0020-2 (PMC6220239; doi:10.1038/s41539-018-0020-2)
Supplement: Supplementary file 1 — supplemental material(DOCX 2707 kb) [file 41539_2018_20_MOESM1_ESM.docx]

**Supplemental Tables**

**Supplemental table 1** Bayesian estimates of bias and inflation from the EWAS analyses.

**Supplemental table 2** Meta-analysis summary statistics for 58 CpG sites significantly associated with educational attainment.

**Supplemental table 3** Number of methylation sites significantly associated with educational attainment level in each analysis.

**Supplemental table 4** Correlation between smoking status and educational attainment.

**Supplemental table 5** Summary statistics based on analysis in the Netherlands Twin Register adjusting for additional smoking phenotypes.

**Supplemental table 6** Association between maternal smoking and methylation level at education top sits in the Netherlands Twin Register, adjusted for individual smoking status

**Supplemental table 7** Results from the association analysis of methylation level and transcript levels in cis (< 100kb).

**Supplemental table 8** Correlation between fetal age and DNA methylation level in the brain from Spiers et al (2015)^1^ for CpGs significantly associated with educational attainment and with a significant change in methylation in fetal brains.

**Supplemental table 9** Correlation between DNA methylation level in blood and DNA methylation in 4 brain regions from Hannon et al (2015)^2^ for CpG sites significantly associated with educational attainment and significantly correlated across blood and brain.

**Supplemental Figures**

**Supplemental Figure 1 Cohort trends in educational attainment level.** a) Each biobank is plotted separately. The pink line shows the mean trend for women and the blue line shows the mean trend for men. b) Women (all biobanks combined). C) Men (all biobanks combined).

A)


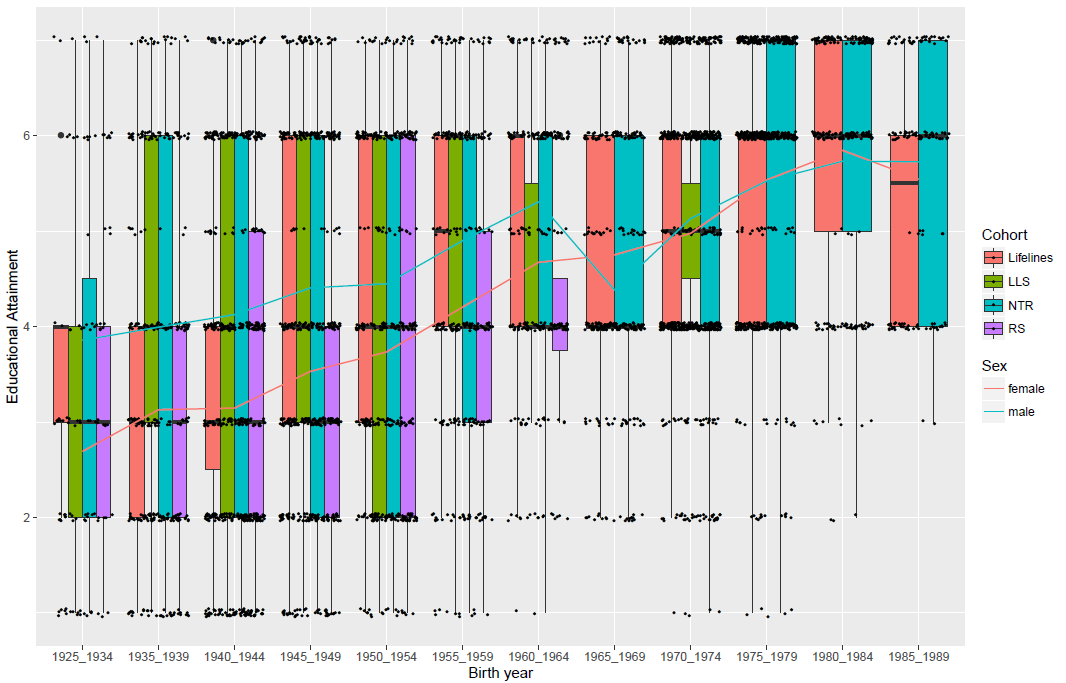


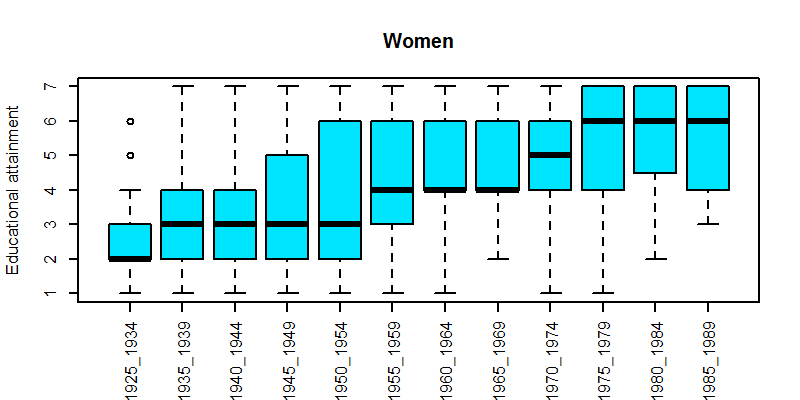

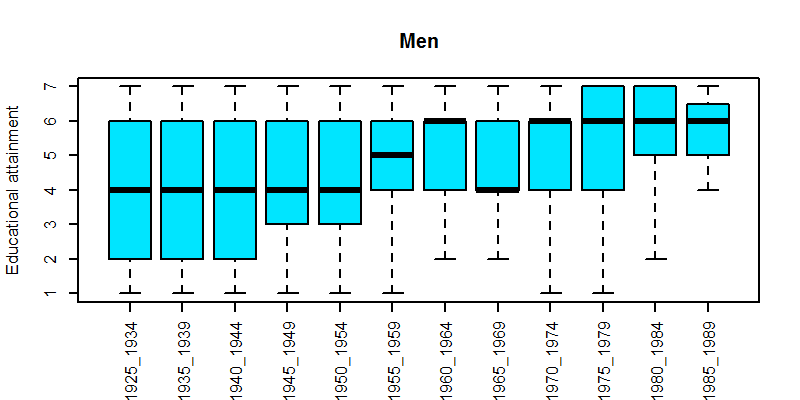
B) C)

**Supplemental Figure 2** Birth cohort- and sex-specific educational attainment ridit scores plotted against educational attainment level **.** a) women b) men.

**Supplemental figure 3: Forest plot showing the association between cg05575921 methylation level and educational attainment in each cohort and in the meta-analysis.**

**
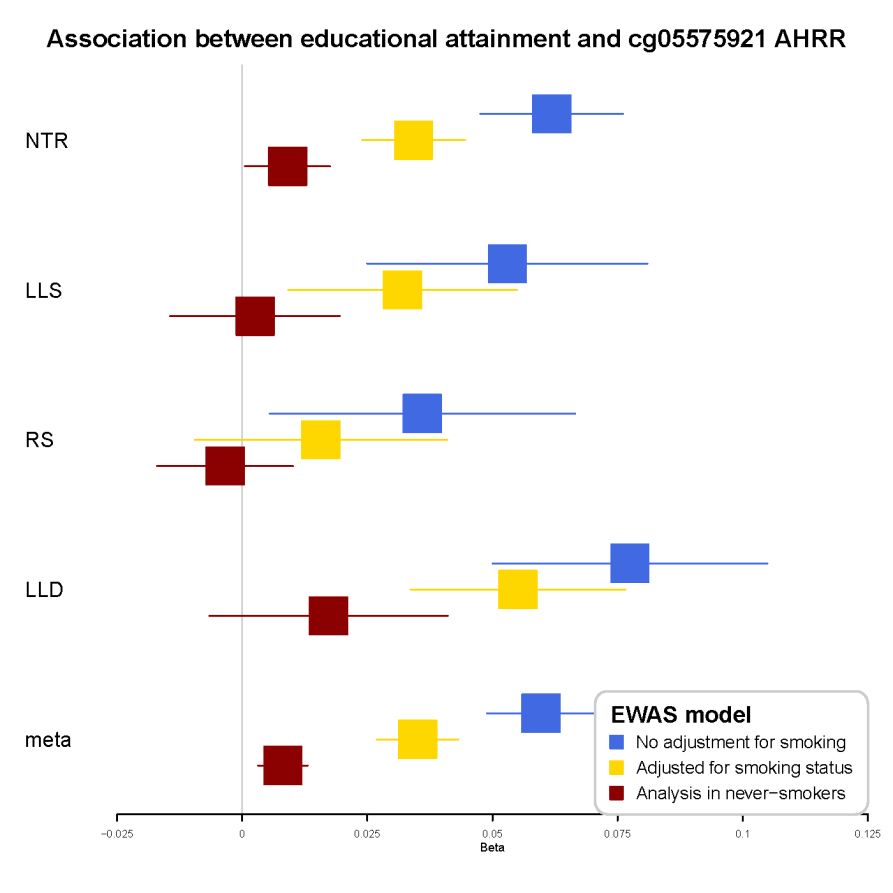
**

**Supplemental figure 4. Power analysis results for educational attainment effect sizes in never smokers.**


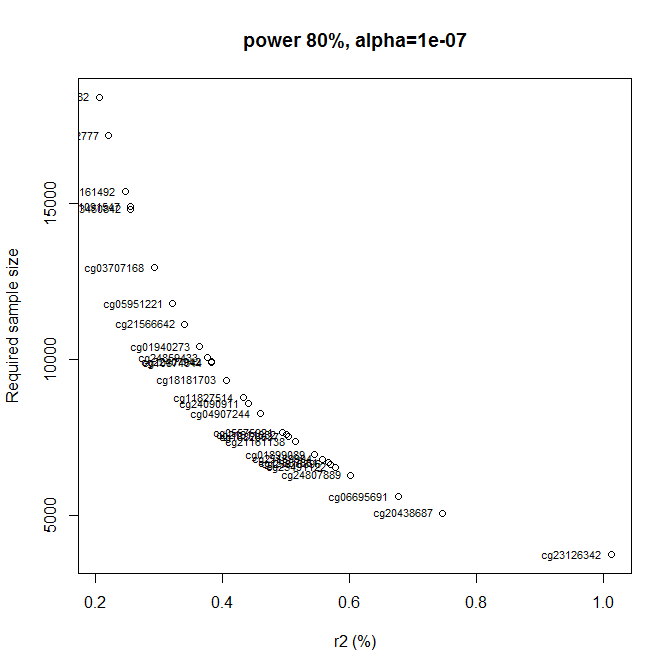


The figure shows the sample size (y-axis) required to achieve 80% power to detect CpGs at genome-wide significance after Bonferroni correction (alpha= 1 x 10^-7^), based on their effect size (r^2^) derived from the current meta-analysis of never smokers (x-axis). The power analysis was performed for the 58 top sites of educational attainment. Only sites with r^2^ > 0.2 in never smokers are displayed in this figure.

**
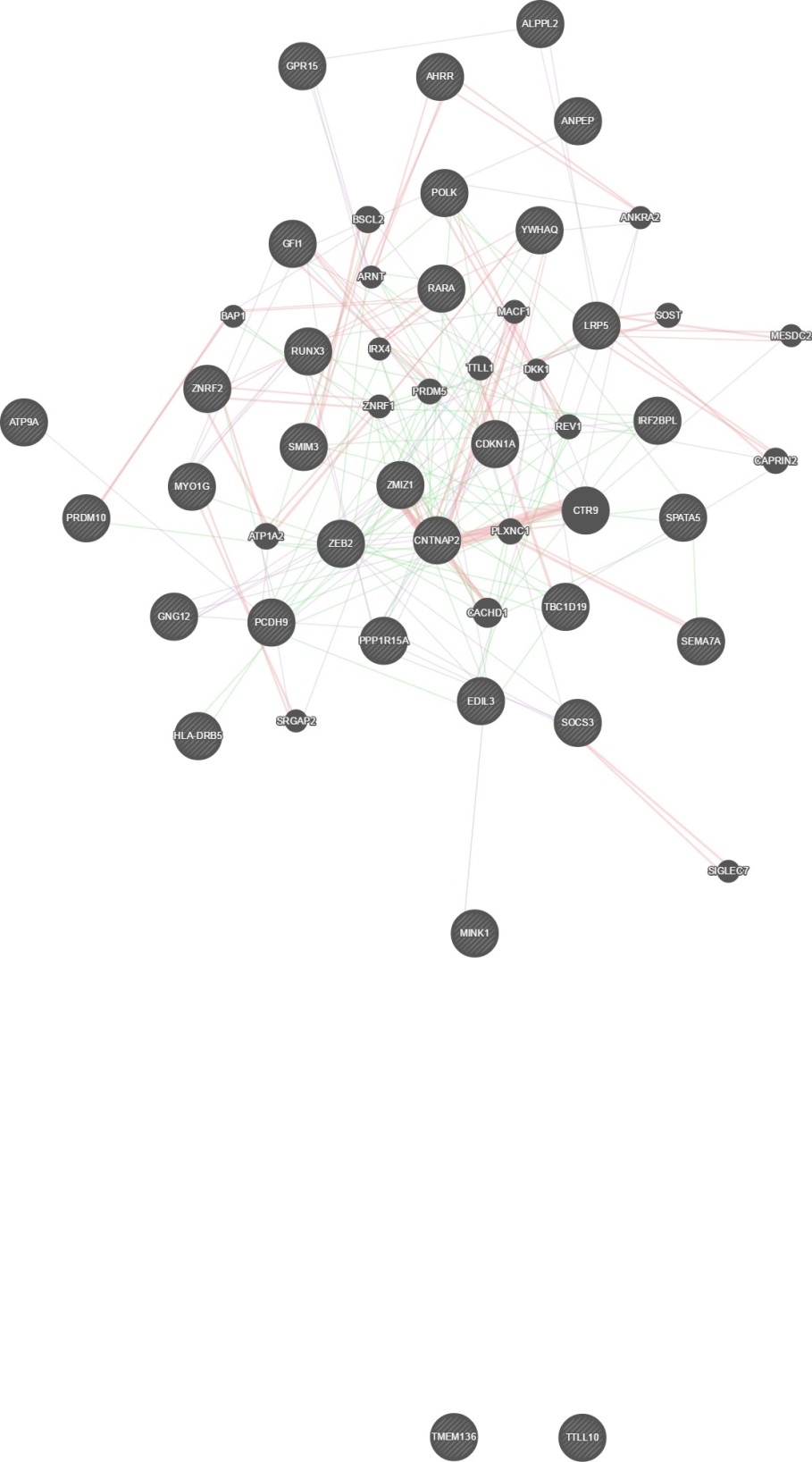
Supplemental figure 5 Network analysis of genes annotating to 58 CpG sites associated with educational attainment.** Input genes are indicated with stripes. Neighbouring genes part of the network but not part of the query genes are indicated by circles without stripes. Red=physical interaction. Green=Genetic interaction. Yellow=shared protein domain. Blue=pathway. Purple=co-expression. Blue=co-localization. A) Network of (nearest) genes of 58 CpG sites associated with educational attainment. B) Network of genes in cis (< 100kb) of education-associated CpGs whose transcript levels correlate with methylation level of education-associated CpGs in blood. C) Network of genes that harbour a CpG site associated with educational attainment of which the methylation level is not significantly associated with the level of any transcripts in cis in blood.

**A**

**B**
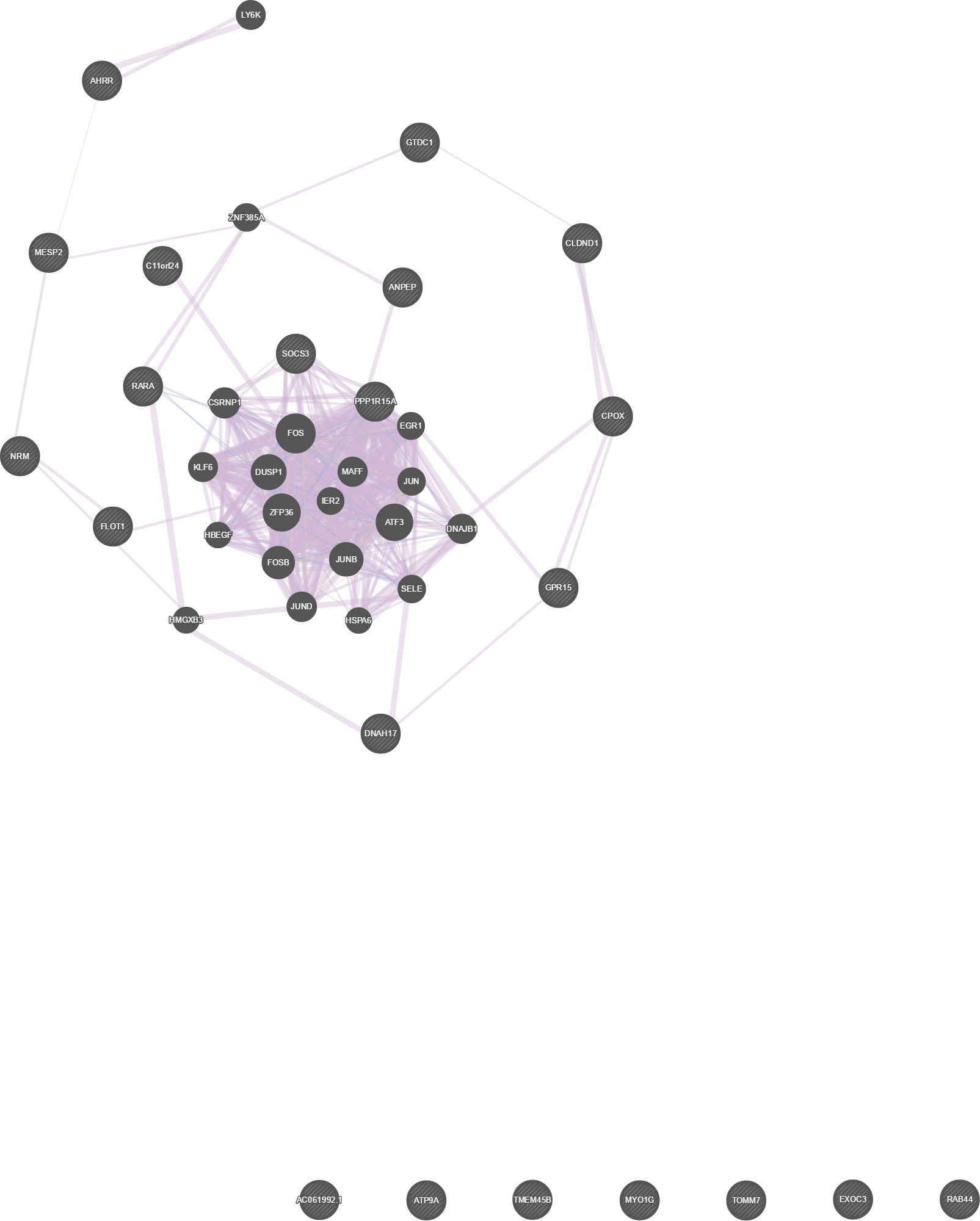


**C**
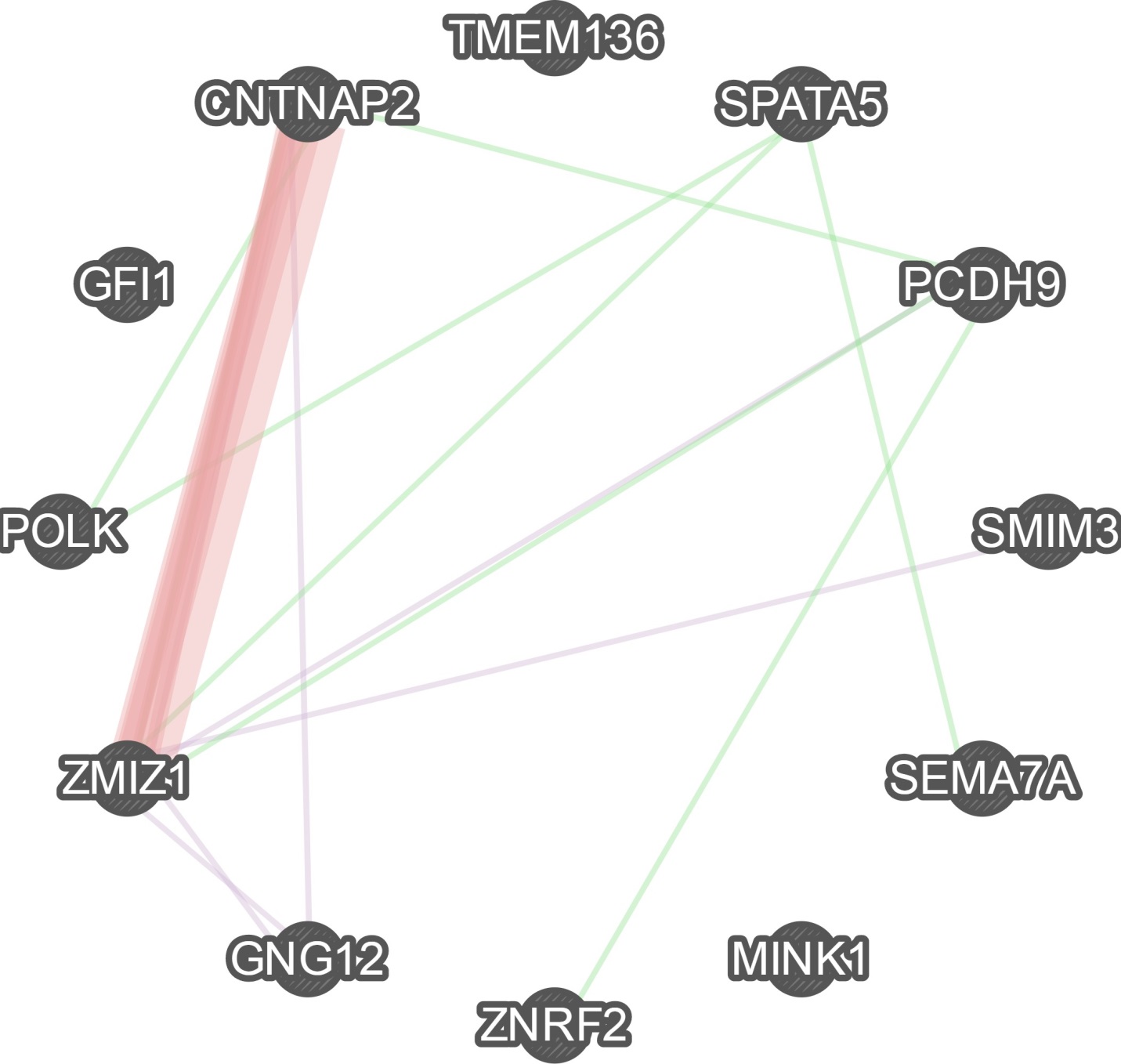


**Supplemental figure 6** Mediation analysis.


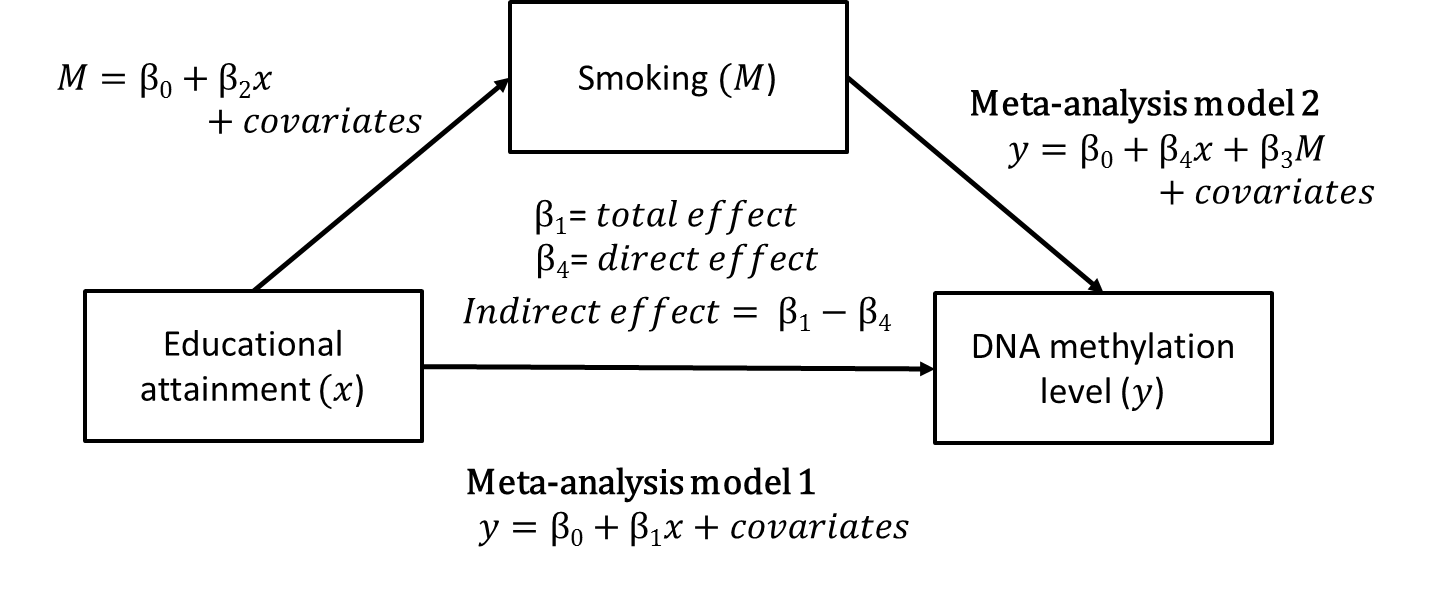


Schematic representation of mediation model. To examine how much of the association between DNA methylation and educational attainment is mediated by smoking status, the estimates (betas) from meta-analysis model 1 (without smoking as covariate) and meta-analysis model 2 (with smoking as covariate) were used to compute the indirect effect (effect mediated by smoking): indirect effect= total effect (β1= beta for educational attainment from meta-analysis model 1) minus direct effect (β4= beta for educational attainment from meta-analysis model 2). The proportion of effect mediated by smoking was computed as: indirect effect/total effect.

**Supplementary Note 1**

**BIOS Consortium (Biobank-based Integrative Omics Study)**

**Management Team** Bastiaan T. Heijmans (chair)^1^, Peter A.C. ’t Hoen^2^, Joyce van Meurs^3^, Aaron Isaacs^4^, Rick Jansen^5^, Lude Franke^6^.

**Cohort collection** Dorret I. Boomsma^7^, René Pool^7^, Jenny van Dongen^7^, Jouke J. Hottenga^7^ (Netherlands Twin Register); Marleen MJ van Greevenbroek^8^, Coen D.A. Stehouwer^8^, Carla J.H. van der Kallen^8^, Casper G. Schalkwijk^8^ (Cohort study on Diabetes and Atherosclerosis Maastricht); Cisca Wijmenga^6^, Lude Franke^6^, Sasha Zhernakova^6^, Ettje F. Tigchelaar^6^ (LifeLines Deep); P. Eline Slagboom^1^, Marian Beekman^1^, Joris Deelen^1^, Diana van Heemst^9^ (Leiden Longevity Study); Jan H. Veldink^10^, Leonard H. van den Berg^10^ (Prospective ALS Study Netherlands); Cornelia M. van Duijn^4^, Bert A. Hofman^11^, Aaron Isaacs^4^, André G. Uitterlinden^3^ (Rotterdam Study).

**Data Generation** Joyce van Meurs (Chair)^3^, P. Mila Jhamai^3^, Michael Verbiest^3^, H. Eka D. Suchiman^1^, Marijn Verkerk^3^, Ruud van der Breggen^1^, Jeroen van Rooij^3^, Nico Lakenberg^1^.

**Data management and computational infrastructure** Hailiang Mei (Chair)^12^, Maarten van Iterson^1^, Michiel van Galen^2^, Jan Bot^13^, Dasha V. Zhernakova^6^, Rick Jansen^5^, Peter van ’t Hof^12^, Patrick Deelen^6^, Irene Nooren^13^, Peter A.C. ’t Hoen^2^, Bastiaan T. Heijmans^1^, Matthijs Moed^1^.

**Data Analysis Group** Lude Franke (Co-Chair)^6^, Martijn Vermaat^2^, Dasha V. Zhernakova^6^, René Luijk^1^, Marc Jan Bonder^6^, Maarten van Iterson^1^, Patrick Deelen^6^, Freerk van Dijk^14^, Michiel van Galen^2^, Wibowo Arindrarto^12^, Szymon M. Kielbasa^15^, Morris A. Swertz^14^, Erik. W van Zwet^15^, Rick Jansen^5^, Peter-Bram ’t Hoen (Co-Chair)^2^, Bastiaan T. Heijmans (Co-Chair)^1^.

1. Molecular Epidemiology Section, Department of Medical Statistics and Bioinformatics, Leiden University Medical Center, Leiden, The Netherlands

2. Department of Human Genetics, Leiden University Medical Center, Leiden, The Netherlands

3. Department of Internal Medicine, ErasmusMC, Rotterdam, The Netherlands

4. Department of Genetic Epidemiology, ErasmusMC, Rotterdam, The Netherlands

5. Department of Psychiatry, VU University Medical Center, Neuroscience Campus Amsterdam, Amsterdam, The Netherlands

6. Department of Genetics, University of Groningen, University Medical Centre Groningen, Groningen, The Netherlands

7. Department of Biological Psychology, VU University Amsterdam, Neuroscience Campus Amsterdam, Amsterdam, The Netherlands

8. Department of Internal Medicine and School for Cardiovascular Diseases (CARIM), Maastricht University Medical Center, Maastricht, The Netherlands

9. Department of Gerontology and Geriatrics, Leiden University Medical Center, Leiden, The Netherlands

10. Department of Neurology, Brain Center Rudolf Magnus, University Medical Center Utrecht, Utrecht, The Netherlands

11. Department of Epidemiology, ErasmusMC, Rotterdam, The Netherlands

12. Sequence Analysis Support Core, Leiden University Medical Center, Leiden, The Netherlands

13. SURFsara, Amsterdam, the Netherlands

14. Genomics Coordination Center, University Medical Center Groningen, University of Groningen, Groningen, the Netherlands

15. Medical Statistics Section, Department of Medical Statistics and Bioinformatics, Leiden University Medical Center, Leiden, The Netherlands

**Supplementary methods**

**Educational attainment ridit scores**

To calculate the corresponding ridit of each education category, the categories are sorted from the lowest to the highest category, and the cumulative frequency of each category is computed. The ridit of education category *j* represents the mid-point of this category on the cumulative distribution:

${Ridit}_{j}=0.5*p_{j}+\sum_{k<j} p_{k}$,

where $p_{j}$= the frequency of category j, and $\sum_{k<j} p_{k}$= the sum of frequencies of all categories below j.

Prior to ridit transformation, the educational attainment data from the four biobanks were combined into one dataset. Ridit scores were computed on the pooled dataset, but separately by sex and birth cohort. Birth cohorts were assigned by grouping birth year into 5-year bins. Because very few subjects belonged to the oldest birth cohort (1925-1929), this birth cohort was pooled with the second birth cohort. Thus, the first cohort covered 10-years (1925_1934).

**Mediation analysis**

To examine how much of the association between DNA methylation and educational attainment is mediated by smoking status, the estimates (betas) from meta-analysis model 1 (without smoking as covariate) and meta-analysis model 2 (with smoking as covariate) were used to compute the indirect effect (effect mediated by smoking): indirect effect= total effect (β1= beta for educational attainment from meta-analysis model 1, supplemental Figure 6) minus direct effect (β4= beta for educational attainment from meta-analysis model 2, supplemental Figure 6). The proportion of effect mediated by smoking was computed as: indirect effect/total effect.

**Power analysis**

Power analysis was performed to estimate the required sample size to detect the associations between methylation and educational attainment in never smokers with 80% power at genome-wide significance following bonferroni correction (alpha= 1 x 10^-7^), given the effect sizes observed in the current study. Power analysis was performed with the function pwr.f2.test() from the R-package pwr . The percentage of variance explained (r^2^) at education top sites was obtained by squaring the correlation (r), which was derived as follows:

$$r=\frac{\beta}{(\frac{sdy}{sdx})}$$

where $\beta$ is the estimate for educational attainment from the meta-analysis of 4 BIOS cohorts in never smokers (outcome = methylation, predictor is educational attainment)

$sdy$ = standard deviation of DNA methylation level residuals in never-smokers (obtained in NTR after adjusting methylation levels for covariates)

$sdx$ = standard deviation of educational attainment in NTR

**Follow-up analyses**

Follow-up analyses of top loci, including relationships with a methylation-based smoking score, pack-years, and prenatal smoking during pregnancy were performed in NTR . In NTR, we also examined the relationship between methylation level and educational attainment within twin pairs. For this analysis, we first adjusted methylation levels for age, sex, white blood cell counts, smoking status and technical covariates. Next, we performed a linear regression analysis to test whether within-pair differences in educational attainment (predictor) were associated with within-pair differences in methylation residuals (outcome) with the R function lm(). We made use of previously published EWA-study summary statistics to compute the Pearson correlation between methylation changes associated with individual smoking^3^, prenatal maternal smoking^4^, maternal plasma folate level^5^, air pollution^6^ and alcohol use^7^ and methylation changes associated with educational attainment. We also made use of two previously published Illumina 450k datasets. The correlations between DNA methylation levels in blood and DNA methylation levels in four brain regions from matched samples (prefrontal cortex, entorhinal cortex, superior temporal gyrus and cerebellum) were obtained from Hannon *et al*^2^. Data on DNA methylation trajectories during fetal brain development were obtained from Spiers *et al*^1^ and used to compute the Pearson correlation between fetal age and DNA methylation level. Network analysis was performed in GeneMANIA with human data and default settings, using all genes annotated to CpGs significantly associated with educational attainment as input (taking the nearest gene for intergenic CpGs).

**References**

1. Spiers, H. *et al.* Methylomic trajectories across human fetal brain development. *Genome Res.* **25,** 338–352 (2015).

2. Hannon, E., Lunnon, K., Schalkwyk, L. & Mill, J. Interindividual methylomic variation across blood, cortex, and cerebellum: Implications for epigenetic studies of neurological and neuropsychiatric phenotypes. *Epigenetics* **10,** 1024–1032 (2015).

3. Joehanes, R. *et al.* Epigenetic Signatures of Cigarette Smoking. *Circ. Cardiovasc. Genet.* **9,** 436–447 (2016).

4. Joubert, B. R. *et al.* DNA Methylation in Newborns and Maternal Smoking in Pregnancy: Genome-wide Consortium Meta-analysis. *Am. J. Hum. Genet.* **98,** 680–696 (2016).

5. Joubert, B. R. *et al.* Maternal plasma folate impacts differential DNA methylation in an epigenome-wide meta-analysis of newborns. *Nat. Commun.* **7,** 10577 (2016).

6. Panni, T. *et al.* Genome-wide analysis of DNA methylation and fine particulate matter air pollution in three study populations: KORA F3, KORA F4, and the normative aging study. *Environ. Health Perspect.* **124,** 983–990 (2016).

7. Liu, C. *et al.* A DNA methylation biomarker of alcohol consumption. *Molecular Psychiatry* (2016). doi:10.1038/mp.2016.192
